# RUNX3 methylation drives hypoxia-induced cell proliferation and antiapoptosis in early tumorigenesis

**DOI:** 10.1038/s41418-020-00647-1

**Published:** 2020-10-28

**Authors:** Sun Hee Lee, Do Young Hyeon, Soo-Hyun Yoon, Ji-Hak Jeong, Saeng-Myung Han, Ju-Won Jang, Minh Phuong Nguyen, Xin-Zi Chi, Sojin An, Kyung-gi Hyun, Hee-Jung Jung, Ji-Joon Song, Suk-Chul Bae, Woo-Ho Kim, Daehee Hwang, You Mie Lee

**Affiliations:** 1 Vessel-Organ Interaction Research Center, VOICE (MRC), Department of Molecular Pathophysiology, College of Pharmacy, Kyungpook National University, Daegu, 41566 Republic of Korea; 2 School of Biological Sciences, Seoul National University, Seoul, 08826 Republic of Korea; 3 School of Life Sciences and Biotechnology, Kyungpook National University, Daegu, 41566 Republic of Korea; 4 Department of Biochemistry, School of Medicine, Institute of Tumor Research, Chungbuk National University, Cheongju, 28644 Republic of Korea; 5 Department of Biological Science, KI for the BioCentury, KAIST, Daejeon, 34141 Republic of Korea; 6 Center for Plant Aging Research, Institute for Basic Science, DGIST, Daegu, 42988 Republic of Korea; 7 Department of Pathology, Seoul National University College of Medicine, Seoul, 03080 Republic of Korea; 8 Present Address: Hochiminh City University of Food Industry, Hochiminh city, Vietnam

**Keywords:** Tumour-suppressor proteins, Cancer microenvironment, Tumour-suppressor proteins

## Abstract

Inactivation of tumor suppressor Runt-related transcription factor 3 (RUNX3) plays an important role during early tumorigenesis. However, posttranslational modifications (PTM)-based mechanism for the inactivation of RUNX3 under hypoxia is still not fully understood. Here, we demonstrate a mechanism that G9a, lysine-specific methyltransferase (KMT), modulates RUNX3 through PTM under hypoxia. Hypoxia significantly increased G9a protein level and G9a interacted with RUNX3 Runt domain, which led to increased methylation of RUNX3 at K129 and K171. This methylation inactivated transactivation activity of RUNX3 by reducing interactions with CBFβ and p300 cofactors, as well as reducing acetylation of RUNX3 by p300, which is involved in nucleocytoplasmic transport by importin-α1. G9a-mediated methylation of RUNX3 under hypoxia promotes cancer cell proliferation by increasing cell cycle or cell division, while suppresses immune response and apoptosis, thereby promoting tumor growth during early tumorigenesis. Our results demonstrate the molecular mechanism of RUNX3 inactivation by G9a-mediated methylation for cell proliferation and antiapoptosis under hypoxia, which can be a therapeutic or preventive target to control tumor growth during early tumorigenesis.

## Introduction

RUNX transcription factors are essential regulators of diverse biological processes, including embryonic development, cell proliferation, differentiation, lineage determination, and apoptosis [1, 2]. There are three RUNX genes in mammals (RUNX1, RUNX2, and RUNX3), which have distinct tissue-specific expression patterns. The RUNXs heterodimerize with a common non-DNA binding core binding factor beta (CBFβ) subunit, which act as both transcriptional activators and repressors [1, 2]. Among the RUNXs, RUNX3 regulates the decision of the cell to progress through the restriction point of the cell cycle by modulation of chromatin structure [3, 4]. Inactivation of RUNX3 dysregulates this important cell cycle check-point and prevents cell cycle arrest and apoptosis. Consistent with this observation, Runx3-targeted deletion in mouse lungs led to lung adenomas and shortened the latency of mutant Kras induced adenocarcinomas [5]. Transcriptional silencing of RUNX3 by hemizygous deletion or increased DNA methylation in the promoter has been reported in about 60% of human gastric cancers [6]. These observations highlight the tumor suppressor function of RUNX3 and indicate that silencing of RUNX3 plays an important role during early stages of tumorigenesis.

Our previous work demonstrated that RUNX3 was downregulated in gastric cancers under hypoxia by epigenetic mechanism involving histone deacetylation and methylation [7]. Hypoxia stimulated gene silencing of *RUNX3* by histone methylation of the RUNX3 promoter region, while it did not affect DNA methylation of RUNX3 promoter or the expression of DNA methyltransferases. We found that histone methylation by G9a histone methyltransferase (HMT) and deacetylation by HDAC1 diminished *RUNX3* expression and inhibited nuclear translocation of RUNX3 during hypoxia. This transcriptional regulation decreased mRNA level of *RUNX3* as well as the protein level in several gastric cancer cell lines, however, not in all tested [7]. For instance, hypoxia inhibited the protein expression of RUNX3 but not the mRNA expression in SNU5 and SNU484 cells. Although we demonstrated the silencing mechanism of *RUNX3* at the transcription level under hypoxia, these cell lines suggest an additional molecular mechanism regulating RUNX3 expression under hypoxia in gastric cancer.

Despite various studies, the underlying molecular mechanisms involved in the regulation of RUNX3 activity remain not fully understood. In the present study, we first demonstrated the molecular mechanism of Lys methylation of RUNX3 under hypoxia in gastric cancer, an unexplored mechanism of posttranslational modification (PTM) for RUNX3 activity. In addition, we showed the distinctive function of G9a as a non-histone protein methyltransferase that directly methylates RUNX3 in hypoxia. This methylation inhibit the transactivation activity of RUNX3 and thereby impairs its tumor suppressive functions. By using integrated analysis of chromatin-immunoprecipitation sequencing (ChIP-seq) and gene expression profiling, we elucidated the mechanistic link between Lys methylation of RUNX3 under hypoxia and transcriptional regulation of genes involved in cell proliferation, cell cycle arrest, and apoptosis at the global level. Our study provides new insights into the regulation of RUNX3 protein stability under hypoxia and has clinical implications for RUNX3 inactivation during early stages of gastric cancer development.

## Materials and methods

### Cell lines and culture

Human embryonic kidney cells (HEK293) and gastric cancer cells (SUN484, SNU5, MKN1, and SNU16) were purchased from the American Type Culture Collection (Manassas, VA, USA) and the Korean Cell Line Bank (KCLB, Seoul Korea) and passaged according to KCLB protocols. Cells were maintained in RPMI-1640 supplemented with 10% fetal bovine serum (HyClone, Logan, UT, USA) under normoxia (21% O_2_) at 37 °C. For hypoxic exposure, cells were cultured in hypoxic chambers (Thermo Scientific, Waltham, MA, USA and Astec, Fukuoka, Japan) to maintain low oxygen tension (1% O_2_, 5% CO_2_, balanced with N_2_). Mycoplasma contamination was tested using e-Myco™ Mycoplasma PCR Detection Kit (iNtRON Biotechnology). No mycoplasma contamination was detected in all cell lines used in this study.

### Western blot analysis

Western blot analysis was performed as previously described [7]. Antibodies specific to human proteins were anti-RUNX3 (ab40278, Abcam), anti-G9a (#3306, Cell Signaling Technology), and β-actin (SC-47778, Santa Cruz), pan-methyl lysine antibody (ab23366, Abcam).

### Real-time PCR analysis

Real-time RT-PCR was performed using specific primers. The PCR program consisted of an initial denaturation step at 95 °C for 10 s, followed by 40 cycles of 95 °C for 5 s and 60 °C for 1 min. See Table S4 for primer sequences used.

### Immunofluorescence

After cells or tissues were fixed with 4% paraformaldehyde, immunofluorescence was performed as described previously [7]. The slides were examined under fluorescence microscopy (Carl Zeiss, AG, Germany).

### Western blot analysis of human gastric tissues

The human gastric tissues specimens were provided from the Seoul National University Hospital. Proteins were extracted from human gastric tumor or normal tissues by tissue homogenization in RIPA buffer (Thermo Scientific). Tissue lysates were subjected to western blot analysis sing anti-RUNX3 (ab40278, Abcam), anti-G9a (#3306, Cell Signaling Technology), and β-actin (SC-47778, Santa Cruz) antibodies. This study was approved by the Institutional Review Board of Seoul National University Hospital (1706-105-860), and informed consent was obtained from each subject.

### Immunoprecipitation

Immunoprecipitation (IP) and western blot analyses were performed as previously described [7]. Briefly, subconfluent HEK293 cells were transfected with plasmids containing pCS4-3Myc-RUNX3 (full-length RUNX3), RUNX3 deletion/point mutants, pEGFP-G9a, pEGFP-G9a SET deletion mutants, and Flag-tagged G9a deletion mutants. Twenty-four hours post-transfection cells were lysed with RIPA and immunoprecipitated with antibodies to Myc.

### In vitro translation and protein interaction

In vitro translation was performed using TnT Quick Coupled Translation System (Promega, #L1170) according to the manufacture’s instruction. Briefly, pCS4-HA-RUNX3, pCS4-HA-G9a, pCS4-HA-G9a-ΔSET plasmids were translated with TnT rabbit reticulocyte lysate using a TnT SP6 RNA polymerase. In vitro translated proteins were electrophoresed in SDS-PAGE gel and western blot analysis was performed with anti-HA antibody (SC-7392, Santa Cruz) to confirm the protein synthesis. Proteins (400 µg) were immunoprecipitated with anti-RUNX3 antibody (ab40278, Abcam) and immunoblotted with anti-HA antibody to check their interaction.

### Fly strains

The *UAS-lz* was provided by Dr. U. Banerjee (University of California, Los Angeles, CA). The fly line glass multimer reporter (*GMR*)-*Gal4* was obtained from the Bloomington Stock Center (Bloomington, IN). *Drosophila* stocks were maintained and cultured by using standard cornmeal-yeast-agar medium at 25 °C. The EP GenExel library used in the screening was obtained from the BioMedical Research Center of the Korea Advanced Institute of Science and Technology.

### HMT assay

G9a activity was measured using the HMT assay reagent kit (Millipore, Billerica, MA, USA), according to the manufacturer’s instructions. For the in vitro methyltransferase assay, the recombinant Runt domain was incubated with the in vitro translated G9a enzyme using 1 μCi [^3^H]-adenosyl-L-methionine (SAM, PerkinElmer, Waltham, MA) as the methyl donor. ^3^H-methylated protein was transferred to a membrane, soaked in scintillation cocktail and radioactivity was measured using a β-counter (PerkinElmer).

### Protein methyltransferase assays and point mutagenesis

Protein purification and in vitro methyltransferase assays with RUNX3 Runt domain and G9a SET proteins were performed as previously described [8]. The Runt domain of hRUNX3 proteins (0, 2, and 8 μM) were incubated in the reaction mixture. Samples were then separated by SDS-polyacrylamide gel electrophoresis and transferred to a polyvinylidene difluoride membrane. The membrane was applied to FLA-5000 (Fuji Film) for radioactive imaging, stained with Ponceau S, and then scanned with LAS-3000 (Fuji Film) for protein quantification. RUNX3 mutants were generated through site-directed mutagenesis (KOD plus polymerase kit; Toyobo) using the pGEX4T-1 vector containing RUNX3 Runt domain. For the double mutants, we used the pGEX4T-1 vector containing the RUNX3_K129A as a template for T173I, V174M, G176R, R178Q, and R178W, and RUNX3_K171A as a template for S118F, R122C, and A126T. All mutations were verified by DNA sequencing.

### Liquid chromatography tandem mass spectrometry (LC–MS/MS) analysis

GST-RUNX3 proteins with 0.5 mM S-adenosylmethionine (NEB, B9003S) were incubated in the presence or absence of G9a SET for 4 h at room temperature. The reaction was stopped by adding 5× SDS loading buffer and boiling at 95 °C for 10 min. The protein bands corresponding to RUNX3 were excised and subjected to LC–MS/MS analysis. The resulting data were preprocessed by PE-MMR to assign the accurate precursor masses prior to the database search [9]. The processed data were then searched against a protein database that contains the NCBI reference sequence of human RUNX3 protein and common contaminants (180 entries) by using MS-GF+ search engine (v10089) [10] with the following parameters in the target-decoy setting: mass tolerance = 20 ppm for precursor ions, semi-tryptic option, a static modification on carbamidomethylation of cysteine (C, +57.0214 Da), and dynamic modifications on oxidation of methionine (M, +15.994915 Da), mono-methylation on lysine and arginine (KR, +14.0156 Da), and di-methylation on lysine and arginine (KR, +28.0313 Da). Peptides with false discovery rate < 1% were identified. For the mono-/di-methylated peptides at K129 and K171 of RUNX3, manual inspection was performed to ensure that the major peak assignments in the MS/MS spectra were matched with theoretical predictions of *b*- and *y*-fragmented ions using a software SpectrumLook (version 1.5.48) [11] with the default parameters.

### Luciferase reporter assay

The pGL3-6xOSE-Luc reporter plasmid, which contains six tandem repeats of the osteoblast-specific core binding sequence [12], was transfected into subconfluent HEK293 cells using Vivamagic Transfection Reagent (Vivagene, Daegu, Korea) according to the manufacturer’s protocol. Luciferase reporter assay was performed as previously described [13].

### Gene information and expression constructs, and siRNAs

The SET domain of hG9a (NM_006709.3, 913–1103 a.a.) and the Runt domain of hRUNX3 (NM_001031680.2, 65–186 a.a.) were expressed in *Escherichia coli* BL21 (DE3) cells. The full-length RUNX3 cDNA, as well as deletion and point mutants, were amplified by PCR and subcloned into pCS4-3HA or pCS4-3Myc plasmids [13]. The pGL3-6xOSE-Luc construct was provided by SCB (Chungbuk National University, Cheongju, Korea). The full-length pEGFP-G9a and the pEGFP-G9a-∆SET mutant were provided by Dr. Martin Walsh (Mt. Sinai School of Medicine, New York, NY, USA). See Table S3 for siRNA sequences used.

### Chromatin immunoprecipitation (ChIP)

SNU484 cells were transfected with RUNX3 expression plasmids (WT, K129R, or K171R mutant) using Lipofectamine 2000 (Invitrogen, Carlsbad, CA, USA). Twenty-four hours post-transfection cells were exposed to hypoxic conditions for 8 h then processed for ChIP experiments. ChIP-seq experiments were performed using iDeal ChIP-seq kit for Transcription Factors (Diagenode SA, Belgium). Briefly, cells (~1.2 × 10^7^ cells for each ChIP experiment) were fixed for 15 min at room temperature with 1% formaldehyde-containing medium. Nuclei were isolated, and the chromatin was sonicated in the mixture of the shearing buffer and protease inhibitor cocktail to an average size of 220 bp. Sonicated chromatin was used for IP by incubation with anti-RUNX3 antibodies (40 μg; ab11905, Abcam) overnight at 4 °C. One percent of the chromatin used for each ChIP reaction was kept as input DNA. Washed protein A-coated magnetic beads (120 μl) were added to each ChIP reaction and reactions were incubated 2 h at 4 °C. The beads were then incubated in 400 μl elution buffer at 65 °C for 4 h to elute immunoprecipitated materials. We performed ChIP-seq assays using chromatin and input controls from three different cell cultures. The ChIP-seq libraries were prepared using DNA SMART^TM^ ChIP-Seq Kit (Clontech Laboratories, USA) and then run on the Illumina sequencer Hi-Seq 2500 (DNA Link, Korea) with 50-bp paired-end reads. ChIP-qPCR experiments were performed using Pierce Agarose ChIP-PCR Kit (26156, Thermo Fisher Scientific, Waltham, MA, USA), according to the manufacturer’s instructions. Briefly, cells were fixed with 1% formaldehyde for 10 min, and the reaction was stopped by adding glycine. Nuclei were isolated and digested with Micrococcal Nuclease for 20 min at 37 °C. Digested chromatin was immunoprecipitated with anti-myc tag antibody (ab9132, Abcam) overnight at 4 °C. Immunoprecipitants were washed three times and eluted after treatment of proteinase K. Immunoprecipitated chromatin was subjected to qRT-PCR using specific primers. See Table S4 for primer sequences used.

### ChIP-seq data analysis

For the read sequences generated from ChIP-seq for each of 12 samples (2 ChIP samples and 2 input DNA samples for WT, K129R or K171R mutant), adapter sequences were trimmed using cutadapt [14] (version 1.12). Remaining reads were then aligned to the human genome (GRCh38) using Bowtie2 [15] (version 2.2.6). PCR or optical duplicate reads, multi-mapped reads, and the reads aligned with mapping quality (MAPQ) < 5 were filtered out using Picard (version 1.85) and Samtools [16] (version 1.3) before peak calling. For each pair of samples (one ChIP sample and its corresponding input DNA sample), we called peaks with *q* value < 0.01 using MACS2 [17] (version 2.1.1) and filtered out the peaks with the number of mapped reads ≤20. DiffBind [18] (version 2.6.5) was used to identify consensus peaks that were consistently detected in at least two pairs of samples among the total 6 pairs (2 pairs for WT, K129R, and K171R) and also to count the number of reads mapped onto these consensus peaks in the individual samples. In each sample, the numbers of reads were converted to counts per million (CPM) based on the library size; and the CPM values were converted to log_2_-CPM values after adding one to the CPM values. For each comparison (K129R or K171R versus WT), log_2_-fold-changes of consensus peaks were calculated as the mean difference of log_2_-read counts. A normalized read density for the neighboring region (±2 kb) of each consensus peak was calculated using SeqMiner [19] (version 1.3.4) with 50 bp window. Each consensus peak was assigned to a genomic region (promoter, 5′-UTR, CDS, 1st intron, other introns, 3′-UTR, or downstream regulatory region) based on the genomic coordinate of its center.

### RNA isolation and microarray analysis

Total RNA was isolated from SNU484 cells expressing WT, K129R mutant, or K171R mutant RUNX3 proteins 8 h after exposure to hypoxia for microarray experiments. Total RNA integrity was checked using a Bioanalyzer 2100 (Agilent, Santa Clara, CA, USA) and all samples were sufficiently good for gene expression analysis with an RNA integrity number > 9. According to the standard Agilent protocols, the RNA was reverse-transcribed, amplified, and then hybridized onto the array (Agilent-039494 SurePrint G3 Human GE v2 8x60k), which includes 62,976 probes corresponding to 23,705 annotated genes. The mRNA levels were measured for two biological replicates for each condition: hypoxia-treated wild type (WT), hypoxia-treated K129R mutant (K129R), and hypoxia-treated K171R mutant (K171R). Log_2_-intensities of the probes were normalized using quantile normalization [20]. To identify differentially expressed genes (DEGs), we then applied an integrative statistical method previously reported [21] to the following comparisons: (1) K129R versus WT; and (2) K171R versus WT. In brief, for each gene, we calculated a T-statistic value using Student’s *t* test and also a log_2_-median-ratio in each comparison. We then estimated empirical distributions of T-statistic values and log_2_-median-ratios for the null hypothesis (i.e., the genes are not differentially expressed) by random permutation experiments of all samples. Using the estimated empirical distributions, for each gene, we computed adjusted *p* values for the observed T-statistic value and log_2_-median-ratio and then calculated the overall *p* value by combining these *p* values using the Stouffer’s method [22]. Finally, we identified DEGs as the ones that have the overall *p* values < 0.01, as well as *t* test *p* values < 0.1, and absolute log_2_-median-ratios > 0.406 (1.33-fold), the mean of 1st and 99th percentile of the empirical distribution for log_2_-median-ratios.

### Enrichment analysis of GO biological processes

The enrichment analysis of gene ontology biological processes (GOBPs) was performed to identify cellular processes enriched by a list of genes using DAVID software [23]. The GOBPs with *p* < 0.05 computed from DAVID were selected as the ones enriched by the genes used.

### Reconstruction of network model

To construct the network model, we first selected the upregulated genes that are annotated with the GOBPs related to cell death [24], involved in the “Apoptosis” in the Kyoto Encyclopedia of Genes and Genomes (KEGG) pathway database [25], or reported to be involved in apoptosis-related processes based on the previous literatures. We then constructed the network model showing interactions among the selected genes and their first neighbors (nodes). The nodes in the network model were arranged according to the activation or repression information obtained from the KEGG pathway database and the previous literatures.

### Mouse models

Animal experiments were carried out with the approval of the KNU Animal Care and Use Committee (KNU 2014-0189). Six-week-old athymic nude mice [BALB/c-nu/nu (18–21 g)] were purchased from SLC Inc. (Japan), and maintained under specific pathogen-free conditions on a standard diet. Males and females were assigned randomly to experimental groups.

### Analysis of mRNA-seq data from TCGA and ACRG gastric cancer cohort

For the TCGA cohort, we collected Fragment Per Kilobase of transcript per Million mapped reads (FPKMs) for 60,483 gene features for 375 stomach adenocarcinoma samples from TCGA genomic data commons (GDC) data portal [26]. Among these genes, we selected the genes with FPKM > 1 in more than 50% of the tumor samples as expressed genes and normalized FPKM values of the genes using the quantile normalization method [20] after converting them to log_2_-(FPKM + 1). For the ACRG cohort, we collected raw files (.CEL files) for 300 gastric cancer samples (GSE62254) [27] from Gene Expression Omnibus database and then normalized the log_2_-intensity using GCRMA [28] (version 2.48.0). To evaluate the correlation of mRNA expression with patient survival, for each gene, we divided the samples into two groups (top and bottom 25% of patients with highest and lowest log_2_-FPKM or log_2_-intensity values, respectively) and evaluated differences in survival curves between the two groups using Gehan–Breslow–Wilcoxon test [29]. Clinical information (tumor stage and survival data) for patients from TCGA and ACRG cohorts were obtained from NCI GDC data portal and Cristescu et al. [27], respectively.

### Somatic mutations of RUNX3

Somatic mutations of RUNX3 relevant to cancers were obtained from TCGA GDAC (https://gdac.broadinstitute.org) and COSMIC database [30]. For each amino acid, a mutation cluster score was calculated as the sum of the numbers of mutations of the amino acid and two neighboring amino acids based on the scan statistics previously reported [31]. Single nucleotide variations (SNVs) with or without amino acid changes (non-synonymous or synonymous mutations) and insertions/deletions (INDELs) were used to calculate the mutation cluster scores. Mutation cluster regions were then determined as the regions within which the maximum nutation cluster score ≥ 3 (*p* < 0.05 by random permutation test) and the number of amino acids with SNV or INDEL ≥ 5.

### TUNEL, cell proliferation, and colony formation assays

SNU484 cells were transfected with lysine mutants (K129R, K171R, or K129R/K171R) RUNX3 and incubated under hypoxia. To assess proliferation, cells were counted based on trypan blue exclusion. The TUNEL assay was performed according to the manufacturer’s instructions (Promega, Madison, WI, USA). Soft agar colony formation assay for 6 days was performed as previously described [32].

### In vivo tumor xenograft experiments and immunohistochemistry (IHC)

Stable cell lines were established by transfecting MKN1 cells (1 × 10^7^) with pEGFP-G9a+pcDNA-3Myc-RUNX3, pEGFP-G9a+pcDNA-3Myc-RUNX3 K129R, pEGFP-G9a+ pcDNA-3Myc-RUNX3 K171R, pEGFP-G9a+ pcDNA-3Myc-RUNX3 K129/171R vector and then selected with G418. The stable cells were mixed with Matrigel (BD Biosciences) and subcutaneously injected into both sides of the mouse flank. Tumor size was measured every other day from 10 days after injection to the 26th day. IHC against CD31 was performed on frozen sections (10 µm) of tumor tissues to determine microvessel density. TUNEL assay was performed with CD31 immunostaining to detect cell apoptosis, and Ki67 immunostaining was performed to visualize proliferating cells. For detection of hypoxic regions in a tumor mass, Hypoxyprobe-1™ (60 mg/kg, Natural Pharmacia International) was injected at 90 min before tissue fixation. Tumors were harvested, sectioned, and stained with Alexa Flour 647-conjugated anti-Hypoxyprobe antibody and anti-Runx3 (D234-3, MBL International) or anti-G9a antibody (#3306, Cell Signaling Technology), followed by Alexa Flour 488-conjugated IgG. Tumor volumes (cm^3^) were calculated by multiplying height by length by depth.

### Tissue microarray experiments

Surgically resected gastric cancer tissues from 450 gastric cancer patients were obtained during surgery at Seoul National University Hospital. To investigate RUNX3 protein expression in these tissues, we generated tissue array slides and then immunostained them using anti-RUNX3 antibody (Active Mofit, #39301; MBL International #D234-3) using Vectastain Elite ABC peroxidase kit (#PK-6100, Vector Laboratories, Burlingame, CA). Briefly, sections (4 µm) were cut from each tissue array block, deparaffinized with xylene and dehydrated using ethanol. After antigen retrieval with 10 mM sodium citrate buffer, endogenous peroxidase activity was quenched with 0.6% H_2_O_2_. Normal serum was used to block nonspecific protein binding. IHC staining for RUNX3 was performed using a standard avidin-biotin immunoperoxidase complex method (Vectastatin Elite ABC peroxidase kit: Vector Laboratories, Burlingame, CA). The immunostaining interpretation was graded as 0, 1, 2, or 3 based on the intensity of the staining. Clinicopathologic parameters, such as age, sex, histologic subtype, presence of lymphatic invasion, depth of invasion, presence of lymph node or distant metastasis, and pathologic stage were evaluated by reviewing the medical charts and pathology records. The clinical outcome was determined from the date of surgery until death or up to 60 months. The data from cases lost to follow-up and deaths caused by problems other than GC were censored during the survival analysis. This study was approved by the Institutional Review Board of Seoul National University Hospital (1911-120-1080), and informed consent was obtained from each subject.

### Statistical analysis

Details of statistical analyses are indicated in the figure legends or described in the “Materials and methods” section. Statistical analyses were performed using GraphPad Prism v7.04 or MATLAB R2016a. Student’s *t* test or ANOVA followed by multiple comparisons testing were used to compare experimental groups as indicated in the figure legends. The number of independent replicates for each experiment is indicated in the figure legends.

### Accession numbers

The microarray and ChIP-seq data generated in this study have been deposited in NCBI Gene Expression Omnibus database (accession numbers of GSE81693 for microarray data and GSE124481 for ChIP-seq data).

## Results

### G9a physically and genetically interacts with RUNX3

**Fig. 1:**
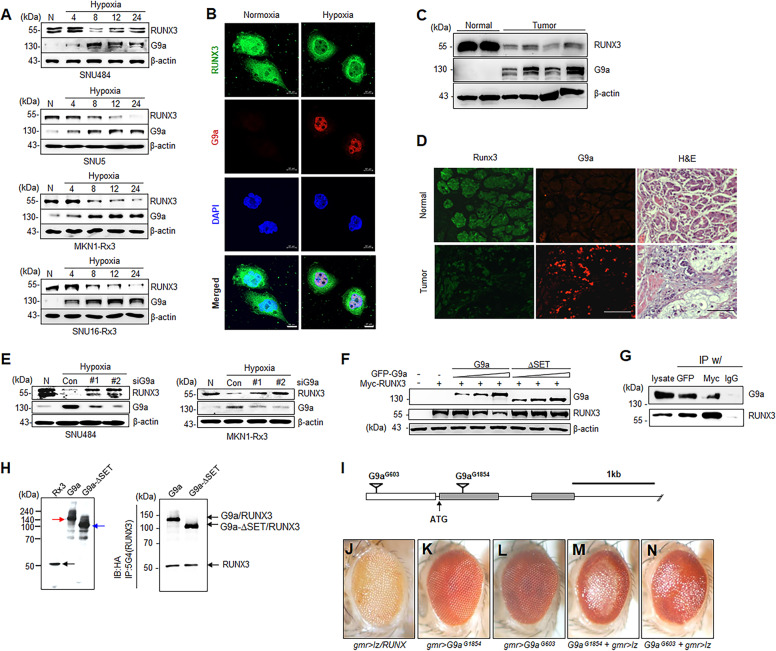
G9a physically and genetically interacts with RUNX3. **A** Western blot analysis of G9a and RUNX3 expression under normoxia (N) and hypoxia for 4, 8, 12, and 24 h in SNU484, SNU5, MKN1-Rx3, and SNU16-Rx3 cells. **B** Immunofluorescence of RUNX3 and G9a expression under normoxia and hypoxia for 8 h using a confocal laser scan microscope. Nuclear DNA was detected by staining with 4′,6-diamidino-2-phenylindole (DAPI). Scale bar = 10 μm. **C** Western blot analysis of G9a and RUNX3 expression in human gastric normal and tumor tissues. **D** Immunofluorescence analysis of RUNX3 and G9a expression in human gastric cancer tissue section. Nuclei were counterstained with Harris-modified hematoxylin. Scale bar = 100 μm. **E** Western blot analysis of RUNX3 and G9a expression under hypoxia for 8 h in SNU484 and MKN1-Rx3 cells transfected with scrambled (Con-si) or two different G9a siRNAs (siG9a #1 and #2). **F** Western blot analysis of RUNX3 in MKN1 cells transfected with wild-type G9a (pEGFP-G9a) or G9a-∆SET (pEGFP-G9a-∆SET). **G** Co-immunoprecipitation (IP) assay of RUNX3 and G9a in HEK293 cells transfected with pCS4-3Myc-RUNX3 or pEGFP-G9a. Whole-cell lysates and immunoprecipitates were analyzed by immunoblotting with anti-G9a or anti-RUNX3 antibodies. **H** In vitro translated proteins (20 µg) of RUNX3, G9a, and G9a-ΔSET were electrophoresed in SDS-PAGE gel and performed by western blot analysis with anti-HA antibody (left panel). Black arrow indicates RUNX3 protein, red arrow indicates G9a protein, and blue arrow indicates G9a-ΔSET protein. In vitro translated proteins (400 µg) of RUNX3 and G9a or G9a-ΔSET were mixed, immunoprecipitated with anti-RUNX3 antibody and immunoblotted with anti-HA antibody (right panel). **I** P-element insertion sites of two loss-function mutants G603 and G1854 in the genomic structure of G9a. ATG, translation initiation codon. **J**–**N** Dissecting stereomicroscope images of ommatidial defects in the indicated mutant lines.

We examined protein expression of G9a and RUNX3 under hypoxia in gastric cancer cell and investigated the patterns of exogenously expressed RUNX3 under hypoxia. Protein levels of G9a were increased under hypoxia, while protein levels of RUNX3 were decreased under hypoxia in the gastric cancer cell lines (Fig. 1A) without affecting mRNA levels of RUNX3 (Fig. S1A, B). We next examined the expression and localization of RUNX3 and G9a in vitro under hypoxia using immunoflurosescence (IF). RUNX3 expression was decreased and localized in the cytoplasm under hypoxia, while G9a expression was increased and localized in the nucleus under hypoxia (Fig. 1B). To examine the time point when G9a binds with RUNX3, we first tested the binding for various periods of time under hypoxia (less than 1% O_2_) using IP and immunoblotting (IB) analysis. We found that the binding of G9a with RUNX3 was highly increased under hypoxia after 8 h in SNU484 cells (Fig. S1C), which interaction was mostly observed in the nucleus of SNU484 cells (Fig. S1D). The estimated proportion of their binding in the cytoplasm and the nucleus under hypoxia was 1.0–7.6, respectively (Fig. S1E). We confirmed the localization of RUNX3 either in normoxia (20% O_2_) or hypoxia (1% O_2_) using α-tubulin as a cytosolic marker and DAPI as a nuclear marker. RUNX3 was co-localized either with DAPI in the nucleus or with α-tubulin in the cytoplasm under normoxia (Fig. S1F). Most RUNX3 was distributed in the cytoplasm and co-localized with α-tubulin in the cytoplasm under hypoxia while a little RUNX3 was co-localized with DAPI in the nucleus (Fig. S1F). These results indicate that most RUNX3 was translocated to the cytoplasm under hypoxia for 8 h. In addition, RUNX3 protein expression was decreased while G9a expression was increased in human gastric tumor tissue compared to normal human gastric tissue (Fig. 1C). Consistently, IF analysis of human gastric tumor tissue sections showed that gastric cancers had low expression of RUNX3 compared to the normal, while gastric cancers had high expression of G9a compared to the normal (Fig. 1D), and this pattern was also seen in various human cancers (Fig. S1C). These results suggest that the reciprocal expression between G9a and RUNX3 is manifested in various human cancers.

Knockdown of G9a by siRNA restored the protein levels of RUNX3 downregulated by hypoxia (Fig. 1E). G9a overexpression by WT G9a plasmid downregulated the protein levels of RUNX3 in a dose-dependent manner (Fig. 1F), whereas a mutant G9a with the SET domain deleted (G9a-∆SET) did not alter RUNX3 protein levels. To test the interaction between G9a and RUNX3, G9a (GFP-G9a) was co-expressed with RUNX3 (Myc-RUNX3), and binding was examined by IP. The results show that G9a coprecipitates with RUNX3 and vice versa suggesting an interaction between these proteins (Fig. 1G). To confirm this result, G9a and RUNX3 were translated in vitro and used for an interaction assay. Interestingly, RUNX3 bound with both WT G9a and G9a-∆SET (Fig. 1H), which suggested RUNX3 did not directly bind to SET domain of G9a. These results indicate that RUNX3 is reciprocally regulated by G9a through direct binding.

Next, we examined the genetic interaction of drosophila RUNX (*lz/RUNX)* and *G9a* in ommatid development. *lz/RUNX* overexpression resulted in an ommatidial defect that appeared as a rough eye phenotype (Fig. 1J). A large-scale genetic screen using a previously reported fly mutant library including ~20,000 mutants [5] revealed two loss-of-function mutants in G9a, G^603^ and G^1854^, which map to an upstream regulatory region and the 1st exon of G9a, respectively (Fig. 1I). Both of these mutations alone showed no apparent ommatidial defects (Fig. 1K, L) however the combination of these G9a mutants with overexpression of *lz/RUNX* showed a more severe ommatidial defects (Fig. 1M, N) than *lz/RUNX* overexpression in a WT background (Fig. 1J). These results indicate a genetic interaction between *G9a* and *lz*/*RUNXs* and further suggest that G9a negatively regulates RUNX3 in the fly ommatid.

### G9a methylates RUNX3 by direct binding

**Fig. 2:**
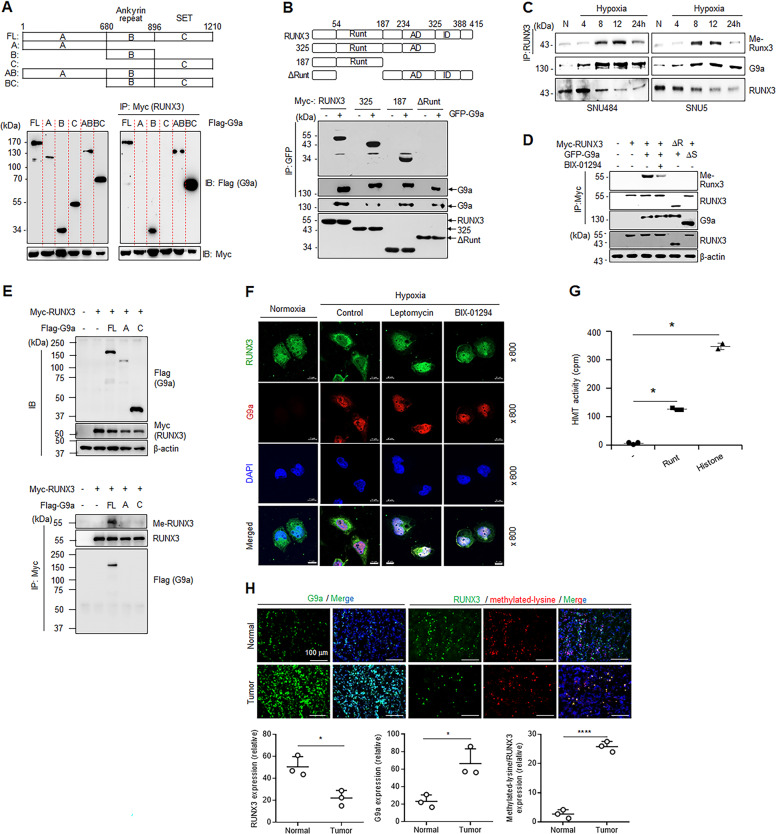
G9a mediates RUNX3 methylation under hypoxia. Schematic diagram showing full and truncated constructs of G9a (A, B, C, AB, and BC (**A**) or RUNX3 (full-length RUNX3, truncated RUNX3 at 325 or 187, and ΔRunt (**B**) (upper panel) and interactions of the constructs with G9a or RUNX3 measured in HEK293 cells (lower panel). **C** Western blot analysis of methylated RUNX3 (Me-RUNX3) in SNU484 and SNU5 cells under normoxia (N) and at 4, 8, 12, and 24 h after hypoxic exposure. **D** Western blot analysis of methylated RUNX3 (Me-RUNX3) in HEK293 cells treated with vehicle or BIX-01294 or transfected with wild-type or mutant RUNX3 or G9a. ΔR, ΔRUNT; ΔS, ΔSET. **E** Western blot analysis of G9a and RUNX3 in HEK293T cells co-transfected with G9a plasmids (pEGFP-G9a; FL, A, and C) or RUNX3 plasmid (pCS4-3Myc-RUNX3). β-actin was used as a loading control (upper). Co-immunoprecipitation (IP) assay and western blot analysis of methylated lysine for methylated RUNX3 (Me-RUNX3) in the HEK293 cells (lower). **F** Immunocytochemistry analysis of RUNX3 (6E9 antibody) and G9a in SNU484 cells treated with vehicle, BIX-01294 (10 µM), or leptomycin (10 µM) for 16 h under hypoxia. Nuclear DNA was detected by staining with 4′,6-diamidino-2-phenylindole (DAPI). Scale bar = 10 μm. **G** In vitro methyltransferase assays of recombinant Runt and histone proteins. Data are shown as the mean ± SD (*n* = 2-3). cpm counts per minute; *p < 0.05 by one-way ANOVA with Tukey’s posthoc corrections. **H** Immunofluorescence analysis of G9a, RUNX3, and methylated-lysine expression in human gastric normal and tumor tissues. Nuclei were counterstained with DAPI. Scale bar = 100 μm. *p < 0.05; ****p < 0.0001 by one-way ANOVA with Tukey’s posthoc corrections.

To further elucidate the interaction between G9a and RUNX3, we sought to determine the specific location where G9a bound to RUNX3 using domain mapping analysis. For this, we made Flag-tagged mutants of G9a that correspond to the three conserved domains in G9a (Fig. 2A, upper panel) [33–35]. RUNX3 preferentially bound to the G9a mutant corresponding to the central ANK (domain B) in G9a (Fig. 2A, lower panel), which is known to be involved in protein–protein interactions [34, 36]. To further determine the specific location of RUNX3 binding to G9a, Myc-tagged mutants of RUNX3 were used. RUNX3 has three conserved domains (Fig. 2B, upper panel) [37]. G9a did not bind to RUNX3-∆Runt (Fig. 2B, lower panel), which is missing the conserved DNA-binding domain (128aa) that binds to a specific motif in DNA [37]. Together these data suggest that the ankyrin repeat region of G9a interacts with the Runt domain of RUNX3.

Since G9a also plays a role as a non-histone protein methyltransferase [38, 39], we examined whether G9a methylated RUNX3 under hypoxia. RUNX3 methylation was increased by hypoxia in a time-dependent manner (Fig. 2C), suggesting G9a could methylate RUNX3 under hypoxia. To examine the RUNX3 methylation by G9a, GFP-G9a was co-overexpressed with Myc-RUNX3, and the levels of RUNX3 methylation was measured. The co-overexpression with the WT plasmids also induced RUNX3 methylation (Fig. 2D). Treatment of the cells with a specific inhibitor of G9a methyltransferase, BIX-01294 [40], reduced methylation of RUNX3 (Fig. 2D). In addition, deletion of the Runt domain in RUNX3 (∆R) or SET domain in G9a (∆S) did not induce RUNX3 methylation. We also examined the RUNX3 methylation with the constructs A or C of G9a, which do not contain region B. The expression of G9a or RUNX3 was confirmed using western blot analysis in HEK293 cells co-transfected with G9a plasmids (pEGFP-G9a; FL, A, and C) or RUNX3 plasmid (pCS4-3Myc-RUNX3). We found that the constructs of RUNX3 or G9a was co-expressed in the HEK293 cells (Fig. 2E, upper). We next examined the binding interaction of RUNX3 with G9a using IP assay, and we found that FL construct induced RUNX3 methylation, while A or C constructs did not (Fig. 2E, lower). These results indicated that G9a without region B was not able to methylate RUNX3. The expression and localization of RUNX3 was examined by treatment of BIX-01294 or leptomycin using IF. BIX-01294 or leptomycin induced RUNX3 expression and localization in the nucleus (Fig. 2F), suggesting G9a inhibition can induce nuclear residence of RUNX3. Since G9a interacted with the Runt domain of RUNX3, we next investigated whether G9a could methylate the Runt domain using in vitro methylation assays. The result showed that G9a could methylate specifically the Runt domain of RUNX3 (Fig. 2G). To elucidate the RUNX3 methylation by G9a in human biopsy, we compared the expression of RUNX3 and localization of methylated-lysine in human gastric tumor tissues and normal tissues using IF. Although levels of RUNX3 were decreased in gastric tumors, the colocalization of methylated-lysine with RUNX3 was increased in human gastric tumor compared to normal tissues (Fig. 2H). These results suggest that G9a methylates RUNX3 by direct binding both in vitro and in vivo.

### G9a methylated RUNX3 at K129 and K171

**Fig. 3:**
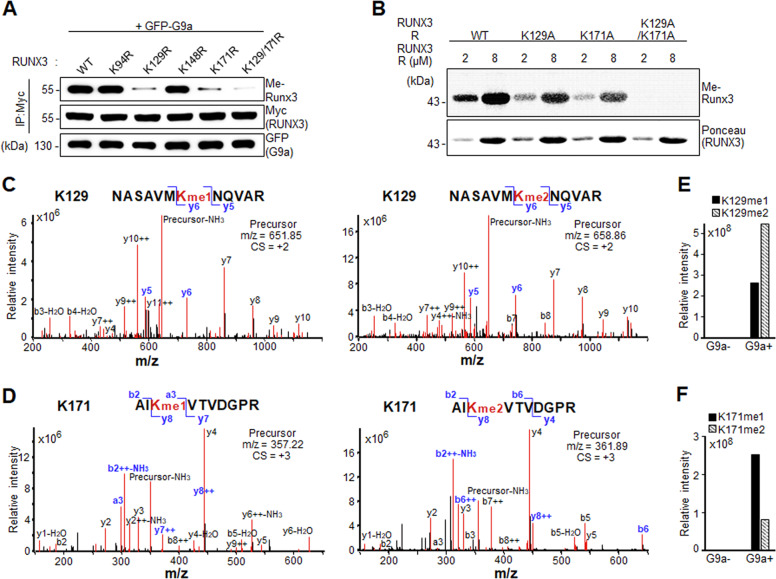
G9a methylates RUNX3 at K129 and K171. **A** Western blot analysis of methylated RUNX3 (Me-RUNX3) in HEK293 cells transfected with G9a (GFP-G9a) and indicated RUNX3 plasmids. **B** In vitro methyltransferase assay of WT or lysine-mutant Runt proteins (2 or 8 µM) of RUNX3 using 3H-SAM as a methyl donor, followed by autoradiography or Ponceau staining. MS/MS spectra of mono- and di-methylated peptides (precursors) containing K129 (**C**) or K171 (**D**) using LC–MS/MS analysis. The spectra show intensities (*y*-axis) of fragmented y and b ions (red lines) at their mass-to-charge (*m*/*z*) ratios (*x*-axis). Fragmented ions at K129 or K171 were labeled in blue. yi, *i*-th y ions; bi, *i*-th b ions; −NH_3_ and –H_2_O, NH_3_ and H_2_O adducts; CS charge state; and + and + +, single and double positively changed ions. Intensities of mono- and di-methylated peptides (precursors) containing K129 (**E**) or K171 (**F**) with or without G9a incubation.

Since G9a methylated RUNX3 (Fig. 2C, D, F), the specific lysine residues of RUNX3 were investigated. The Runt domain of RUNX3 contains six lysine residues, four of which (K94, K129, K148, and K171) are exposed on the surface of the protein [41]. We substituted these four lysine residues to arginine (R) in RUNX3 and examined the ability of G9a to methylate these proteins in cells (Fig. 3A) and with purified proteins (Fig. 3B–F). K129R or K171R mutants showed lower RUNX3 methylation compared to WT, and the double mutant (K129/171R) showed no methylation (Fig. 3A). K94R and K148R mutants showed similar levels of methylation compared to WT (Fig. 3A). In in vitro methyltransferase assays, K129A or K171A mutants also showed lower RUNX3 methylation compared to WT, and the double mutant (K129/171A) showed no methylation (Fig. 3B). To further confirm these results, LC–MS/MS analysis of RUNX3 was performed by following tryptic digestion after incubating RUNX3 with G9a. Through MS-GF+ search [42] of the LC–MS/MS data, mono- and di-methylated peptides of RUNX3 at K129 (Fig. 3C) or K171 (Fig. 3D) were identified. These methylated peptides were not detected in the absence of G9a (Fig. 3E, F). These results suggest that G9a methylates RUNX3 specifically at K129 and/or K171 residues of the Runt domain.

### The methylation by G9a reduces RUNX3 transactivation by inhibiting its interaction with CBFβ and p300

**Fig. 4:**
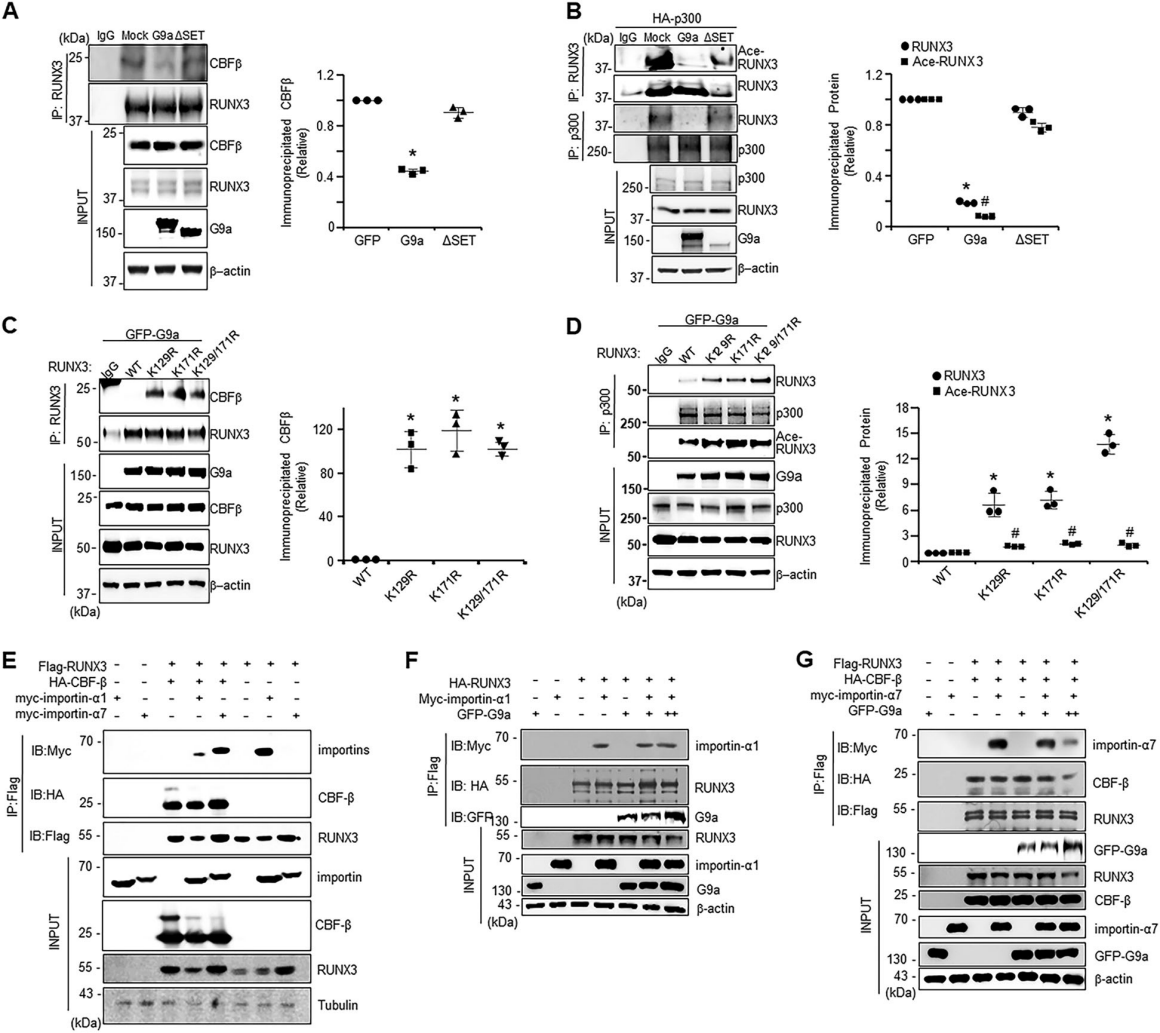
G9a-mediated methylation inhibits interactions between RUNX3 and CBFβ/p300. **A** Co-immunoprecipitation (IP) assay of CBFβ and RUNX3 in HEK293 cells transfected with control vector and G9a plasmids (WT, ∆SET). Immunoprecipitates were analyzed by immunoblotting with anti-CBFβ, anti-RUNX3, anti-G9a, and anti-β-actin antibodies. **B** Co-immunoprecipitation (IP) assay of p300 and RUNX3 in HEK293 cells transfected with control vector, p300 (HA-p300) and G9a plasmids (WT, ∆SET). Immunoprecipitates were analyzed by immunoblotting with anti-acetyl Lysine (#9441, Cell Signaling Technology), anti-RUNX3, anti-p300 (ab10485, abcam), anti-G9a, and anti-β-actin antibodies. **C** Co-immunoprecipitation (IP) assay of CBFβ and RUNX3 in HEK293 cells transfected with G9a plasmid (pEGFP-G9a) and indicated RUNX3 mutants. Immunoprecipitates were analyzed by immunoblotting with anti-CBFβ, anti-RUNX3, anti-G9a, and anti-β-actin antibodies. **D** Co-immunoprecipitation (IP) assay of p300 and RUNX3 in HEK293 cells transfected with G9a plasmid (pEGFP-G9a) and indicated RUNX3 mutants. Immunoprecipitates were analyzed by immunoblotting with anti-RUNX3, anti-p300, anti-acetyl Lysine, anti-G9a, and anti-β-actin antibodies. Protein levels were quantified from the western blot images (*n* = 3), and data are shown as the mean ± SD. *p < 0.05 (CBFβ and RUNX3) and ^#^*p* < 0.05 (Ace-RUNX3) by one-way ANOVA with Tukey’s posthoc correction. **E** Co-immunoprecipitation (IP) assay of RUNX3 and importins in HEK293 cells transfected with RUNX3 (Flag-RUNX3), importins (Myc-importin-α1, Myc-importin-α7), and/or CBFβ (HA-CBFβ) plasmids. Immunoprecipitates were analyzed by immunoblotting with anti-Myc (ab9106, abcam), anti-HA (SC-7392, Santa Cruz), anti-Flag (F1804, Sigma), anti-CBFβ (ab124693, abcam), anti-RUNX3, and anti-Tubulin (SC-5286, Santa Cruz) antibodies. **F** Co-immunoprecipitation (IP) assay of RUNX3 and importin-α1 in HEK293 cells transfected with RUNX3 (Flag-RUNX3), importins (Myc-importin-α1), and/or G9a (pEGFP-G9a) plasmids. Immunoprecipitates were analyzed by immunoblotting with anti-Myc, anti-HA, anti-GFP, anti-RUNX3, anti-importin-α1, anti-G9a, and anti-β-actin antibodies. **G** Co-immunoprecipitation (IP) assay of RUNX3 and importin-α7 in HEK293 cells transfected with RUNX3 (Flag-RUNX3), CBFβ (HA-CBFβ), importin (Myc-importin-α7) and/or G9a (pEGFP-G9a) plasmids. Immunoprecipitates were analyzed by immunoblotting with anti-Myc, anti-HA, anti-Flag, anti-GFP, anti-RUNX3, anti-CBFβ, anti-importin-α7, anti-G9a, and anti-β-actin antibodies.

To examine the role of G9a in the interaction between RUNX3 and CBFβ and p300, the binding of RUNX3 to these proteins was examined with and without overexpression of G9a (Fig. 4A, B). Binding of CBFβ and p300 to RUNX3 was inhibited by overexpression of G9a but not by the G9a-ΔSET mutant. These data indicate that G9a inhibits the formation of heterocomplex RUNX3 with CBFβ and p300. In addition, G9a overexpression decreased the acetylation of RUNX3. These data indicate that G9a inhibits the binding of RUNX3 with p300 and the subsequent acetylation of RUNX3. In contrast the interaction of RUNX3 with CBFβ (Fig. 4C) or p300 (Fig. 4D), as well as acetylation of RUNX3 (Fig. 4D), were significantly increased in RUNX3 mutants (K129R, K171R, and K129/171R) compared to those in RUNX3 WT. These results indicate that G9a-mediated RUNX3 methylation at K129 and K171 inhibits the interaction of RUNX3 with its transcriptional cofactors, CBFβ and p300, and potentially reducing promoter binding of RUNX3.

Importin-α1 strongly bound to RUNX3 in the absence of CBFβ, while weakly bound to RUNX3 in the presence of CBFβ (Fig. 4E). Importin-α7 bound to RUNX3 only when CBFβ was present suggesting importin-α7 does not bind RUNX3 directly (Fig. 4E). To further examine the role of G9a in the formation of the RUNX3 and importin complexes, G9a (GFP-G9a) was co-overexpressed with RUNX3 and the importins. Importin-α1 strongly bound to RUNX3 both in the presence and absence of G9a (Fig. 4F), while the binding of importin-α7 with RUNX3 and CBFβ was inhibited by G9a in a dose-dependent manner (Fig. 4G). These results suggest that RUNX3 is transported into the nucleus in part by binding to CBFβ, which binds importin-α7 and translocates the complex into the nucleus.

### RUNX3 methylation by G9a globally affects RUNX3 target gene expression

**Fig. 5:**
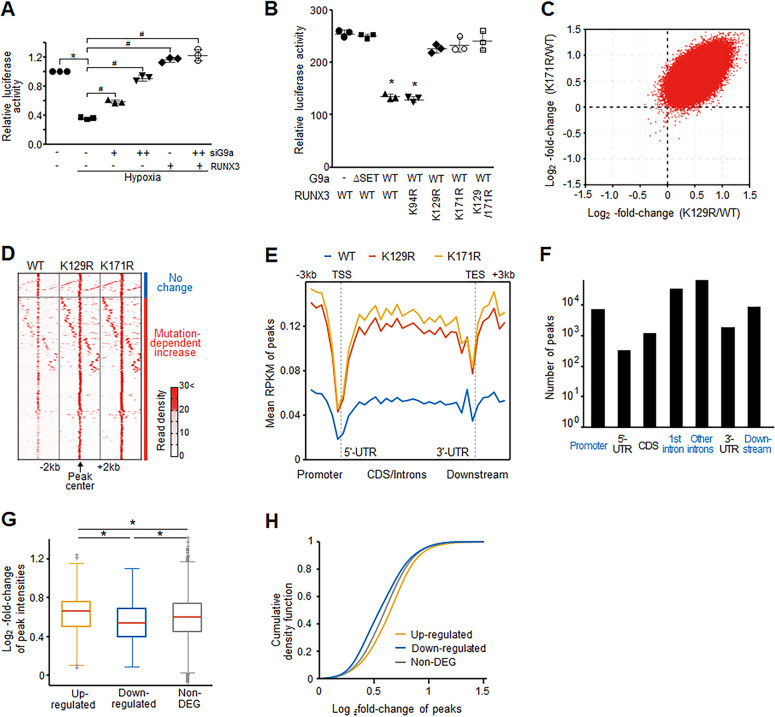
Hypoxia-induced methylation modulates RUNX3 transcriptional activity. **A** Luciferase reporter assay of RUNX3 under hypoxia in HEK293 cells transfected with G9a siRNA (siG9a, +, 5 nM; ++, 20 nM) and/or RUNX3 plasmid (pCS4-3Myc-RUNX3). The result was normalized to the activity of β-gal. Data are shown as the mean ± SD (*n* = 3). *p < 0.05 (normoxia) and ^#^*p* < 0.05 (hypoxia) by one-way ANOVA with Tukey’s posthoc correction. **B** Luciferase reporter assay of RUNX3 in HEK293 cells transfected with G9a plasmids (WT, ∆SET) and/or indicated RUNX3 mutants. The result was normalized to the activity of β-gal. Data are shown as the mean ± SD (*n* = 3). *p < 0.05 by one-way ANOVA with Tukey’s posthoc correction. **C** Scatter plot of log2-fold-changes of consensus peak (red dot) intensities (read counts) for K129R versus WT (*x*-axis) and K171R versus WT (*y*-axis). **D** Distributions of read counts in the neighboring regions of consensus peaks (−2 kb and +2 kb from consensus peak centers). Blue and red bars, consensus peaks with no intensity changes between WT and the mutant and increased intensities in the mutants, respectively. Color bar, gradient of read counts at individual bases. Distribution of the mean consensus peak intensities (RPKMs) (**E**) and numbers of consensus peaks (**F**) in the indicated genomic regions. Distributions (**G**) and cumulative density functions (**H**) of log2-fold-changes of consensus peak intensities in up- and downregulated genes and non-DEGs. *p < 0.05 by one-way ANOVA with Tukey’s posthoc corrections.

Hypoxia reduced the transcriptional activity of RUNX3 and this activity was recovered by G9a knockdown (Fig. 5A). Further, we co-transfected our RUNX3 mutants (K94R, K129R, and K171R) and G9a, and measured the luciferase activity. G9a suppressed the transcriptional activity of RUNX3 WT and RUNX3 mutant (K94R) under hypoxia, while G9a (WT or ΔSET) did not suppress the transcriptional activity of RUNX3 mutant (K129R, K171R) under hypoxia (Fig. 5B). These results indicate that the K129 and K171 in the Runt domain of RUNX3 are essential for the suppression of the transactivation activity of RUNX3 by G9a.

We systematically investigated the modulation of RUNX3 transcriptional activity by G9a-mediated methylation using chromatin-IP sequencing (ChIP-seq). K129R and K171R mutations strongly increased read counts of the consensus peaks (Fig. 5C) and in neighboring regions (Fig. 5D) compared to WT, suggesting an increase of binding affinity of RUNX3 mutants (K129R and K171R) to target genes. The numbers and distributions of the consensus peaks in the genomic regions (Fig. 5E, F) further showed that the binding of RUNX3 was more enriched in the promoter, intron, and downstream regulatory regions than in coding sequences (CDSs) and 5′ and 3′ untranslated regions (UTRs).

To further elucidate the effects of the increased RUNX3 binding in these three enriched regions on mRNA expression of target genes, mRNA expression was profiled in SNU484 overexpressing WT, K129R and K171R mutant RUNX3 under hypoxia. Six hundred and ninety-two DEGs were identified between WT and the mutants, including 387 upregulated and 305 downregulated genes by K129R or K171R mutation. The RUNX3-binding affinity changes were compared in each of the three regulatory regions of the up- and downregulated genes with those of non-DEGs. In the promoter region, the up- and downregulated genes showed significantly (*p* < 0.05) higher and lower log_2_-fold-changes of the consensus peaks than non-DEGs, respectively (Fig. 5G, H). In contrast the intron and downstream regulatory regions of these DEGs showed no significant differences in log_2_-fold-chagnes of the consensus peaks compared to non-DEGs (Fig. S2A–C). Thus, although K129R and K171R mutations increased RUNX3 target promoter binding, they increased the binding strongly and weakly for the up- and downregulated genes, respectively, compared to that for non-DEGs. These results suggest that G9a-mediated methylation decreases RUNX3 target promoter binding with differential extents depending on the target genes.

### RUNX3 methylation promotes cancer cell proliferation but inhibits apoptosis under hypoxia

**Fig. 6:**
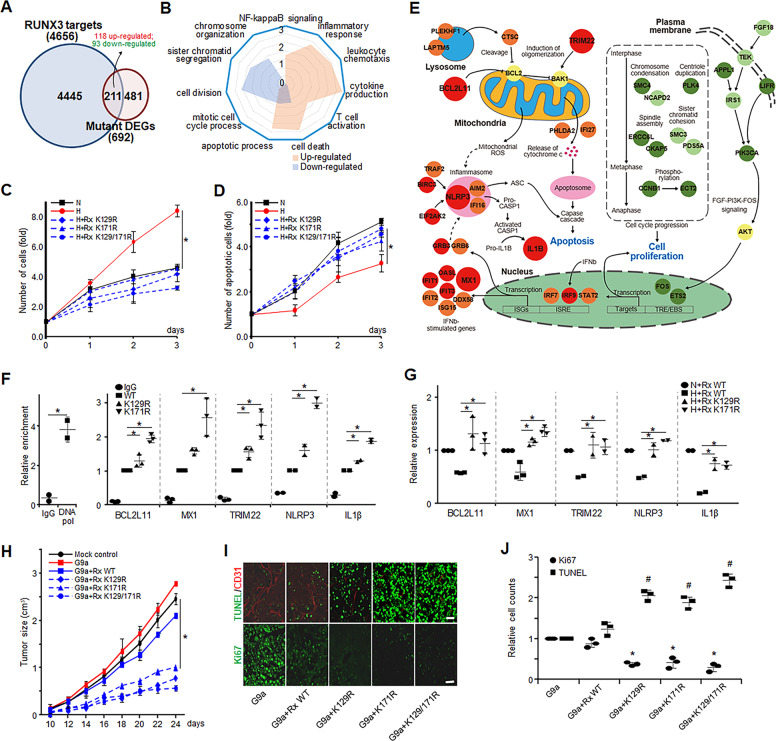
Hypoxia-induced RUNX3 methylation regulates a subset of target genes involved in apoptosis and proliferation. **A** Relationships between DEGs and target genes with differential promoter bindings between RUNX3 WT and mutants. Numbers of DEGs and target genes were denoted in parenthesis. **B** Radar plot showing cellular processes enriched by up- and downregulated genes. Enrichment scores, –log(*p*), where *p* is the enrichment *p* value, for cellular processes. The levels of proliferation (**C**) or apoptosis (**D**) were determined under normoxia (N) or hypoxia (H) by cell count in SNU484 cells stably expressing RUNX3 WT and mutants (Rx K129R, K171R, and K129/171R). **E** Network model describing interactions among up- or downregulated genes involved in cell death/apoptotic process and proliferation, respectively. Node colors, up- (red) and downregulated (green) RUNX3 target genes and genes with no expression changes (yellow) in the mutants, compared to WT. Arrow, activation; suppression symbol, inhibition; solid and dotted lines, direct and indirect interactions, respectively; ISRE interferon (IFN)-sensitive response element, ISGs IFN-stimulated genes, TRE 12-O-Tetradecanoylphorbol-13-acetate (TPA) response element, EBS ETS-binding site. Confirmation of increased promoter binding (**F**) and upregulation (**G**) of five representative genes in SNU484 cells stably expressing WT and mutant RUNX3 (Rx K129R and K171R) by ChIP-PCR and qRT-PCR analyses, respectively. **H** In vivo xenograft assays in nude mice inoculated with MKN1 cells expressing the indicated constructs (*n* = 8 mice/condition). **I** Immunohistochemistry analysis of CD31, Ki67, and TUNEL in the tumors from (**H**). **J** Relative counts of proliferating (Ki67) and apoptotic (TUNEL) cells quantified from the images in (**I**). Cell counts were normalized by those obtained from MKN1 cells expressing G9a. The results in (**C**), (**D**), (**F**), (**G**), and (**J**) represent the mean ± SD of two or three independent experiments. *p < 0.05 and ^#^*p* < 0.05 (TUNEL) by one-way ANOVA with Tukey’s posthoc correction.

We identified 211 DEGs (118 upregulated and 93 downregulated) that also showed increased promoter binding by the mutant RUNX3s (Fig. 6A, Tables S1 and S2). Enrichment analysis of GOBPs revealed that the upregulated genes were mainly associated with the processes related to immune response and apoptosis, whereas the downregulated genes were associated with the processes related to cell cycle (Fig. 6B). These data suggest that RUNX3 methylation by hypoxia promotes gastric cancer cell proliferation through the increased cell cycle or cell division, while it suppresses immune response and apoptosis.

Cell proliferation and colony formation were increased under hypoxia compared to normoxia in the RUNX3 WT, whereas they were significantly (*p* < 0.05) decreased in the K129R, K171R, or K129R/K171R mutants (Fig. 6C and S3A). In addition, the number of apoptotic cells was decreased under hypoxia compared to normoxia in the RUNX3 WT, but not apparently in the mutants (Fig. 6D). The network model showed that both apoptosome and inflammasome-mediated apoptotic pathways were upregulated by the mutations (Fig. 6E). In contrast, the network model showed that the activities of FGF-PI3K-FOS signaling pathway and cell cycle were decreased by the mutations. The target genes were confirmed by ChIP-PCR and qRT-PCR analyses, which are involved in mitochondrial dysfunction (TRIM22), interferon signaling (MX1), and apoptosome (BCL2L11) and inflammasome (NLRP3 and IL1β)-mediated apoptotic pathways (Fig. 6E–G, and S3B).

Since overexpression of RUNX3 alone strongly slowed down tumor growth (Fig. S4), we co-overexpressed G9a with RUNX3 WT or mutants. Tumors expressing K129R (RUNX3 K129R) and/or K171R (RUNX3 K171R) mutants of RUNX3 grew slower than those expressing RUNX3 WT, mock (control), or G9a alone (Fig. 6H). This pattern of tumor growth was similar to that observed with overexpression of RUNX3 WT without G9a (Fig. 6H and S4). In addition, tumors expressing RUNX3 mutants showed significantly (*p* < 0.05) higher numbers of TUNEL-positive cells and lower numbers of Ki67-positive cells than tumors expressing RUNX3 WT (Fig. 6I, J). Taken together, these results suggest that G9a-mediated methylation of RUNX3 under hypoxia enhances cancer cell proliferation by increasing cell cycle or cell division with possibility of suppressing apoptosis through inhibiting inflammasome-mediated apoptotic pathways, which might promote tumor growth.

### RUNX3 methylation has potential clinical implications in human cancers

**Fig. 7:**
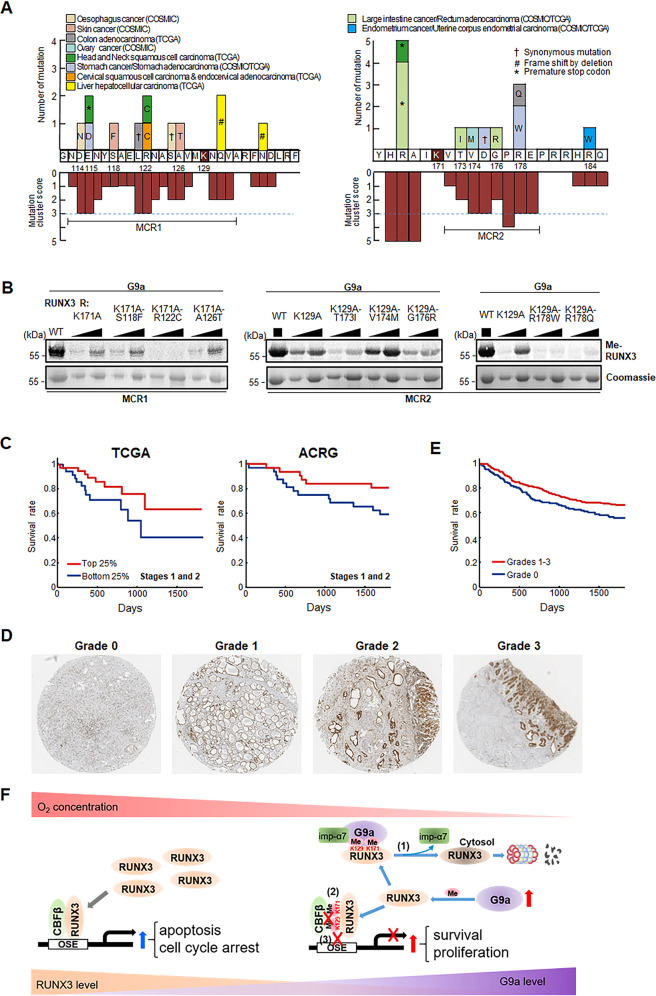
Potential clinical implications of RUNX3 methylation in human cancers. **A** Distributions of non-synonymous somatic mutations of RUNX3 reported in COSMIC database and TCGA data portal near K129 (left) and K171 (right) in indicated cancers. MCRs 1 and 2 with high mutation scores were shown. **B** In vitro methyltransferase assay of RUNX3 in SNU484 cells expressing the indicated RUNX3 constructs and G9a using 3H-SAM as a methyl donor, followed by autoradiography or coomassie staining. **C** Survival differences between two groups of patients expressing high (top 25%) and low (bottom 25%) mRNA expression levels of RUNX3 at stages 1 and 2 in TCGA and ACRG cohorts. **D** Immunohistochemistry analysis of tissue samples in Grades 0–3 from tissue microarray experiments. Brown color represents expression of RUNX3 protein. **E** Overall survival rate in gastric cancer patients with high-grade (Grades 1–3) and low-grade (Grade 0) tumor according to RUNX3 protein expression. **F** A schematic model describing modulation of RUNX3 by G9a-mediated methylation under hypoxia in early gastric tumorigenesis: (1) RUNX3 methylation by G9a under hypoxia and induction of cytosolic localization of RUNX3 via dissociating with importin-α7, (2) inhibition of the RUNX3-CBFβ/p300 interaction, and (3) decrease of RUNX3 binding to the promoter of target genes.

Using somatic mutations of RUNX3 from Catalog of Somatic Mutations in Cancer (COSMIC) database and TCGA data portal [43, 44], two mutation cluster regions (MCR1 and 2) near K129 and K171 (Fig. 7A) were identified. We examined the ability of mutations in MCR1/2 to alter the extent of G9a-mediated methylation at K129 and K171 using in vitro methylation assays. R122C mutation in MCR1 decreased K129 methylation in SNU484 cells expressing K171A mutant. T173I, G176R, and R178W/Q mutations in MCR2 decreased K171 methylation in SNU484 cells expressing K129R mutant (Fig. 7B). These results suggest that somatic mutations can alter the extent of G9a-mediated RUNX3 methylations, thereby inhibiting negative regulation of RUNX3 by G9a in human gastric cancers.

Patients (top 25%) with higher mRNA expression levels of RUNX3 at stages 1 and 2 showed better survival than patients (bottom 25%) with lower mRNA expression levels of RUNX3 (Fig. 7C). However, this survival pattern was not observed in patients at stages 3 and 4 (Fig. S5), consistent with the previously reported role of RUNX3 in early tumorigenesis [3, 4]. To confirm this positive correlation of RUNX3 levels with patient survivals at the protein level, IHC analysis was performed for tissue samples from 450 gastric cancer patients using tissue microarrays. Depending on RUNX3 protein levels in gastric epithelium, the patients were categorized into four groups with Grades 0–3 (Fig. 7D). Patients (Grades 1–3) expressing RUNX3 proteins showed better survival than patients (Grade 0) with virtually no RUNX3 protein expression (Fig. 7E). Our results provide a molecular mechanism for the hypoxia-induced inhibition of RUNX3, which can enhance survival/proliferation of gastric cancer cells and suppressing their apoptosis during early tumorigenesis (Fig. 7F): hypoxia induces G9a that methylates RUNX3 at K129/K171, inducing cytosolic localization of RUNX3 via dissociating with importin-α7 (1) and the methylation decreases the binding of RUNX3 to the promoter of target genes (3) by inhibiting interactions of RUNX3 with CBFβ and p300 (2).

## Discussion

Diverse PTMs affect tumor suppressive functions of RUNX3, such as stability, subcellular localization, and transactivation activity [1]. p300-mediated acetylation of RUNX3 induced by TGFβ signaling inhibits degradation of RUNX3 via Smurf-dependent ubiquitination [45]. In contrast, sumoylation of RUNX3 by PIAS suppresses its transactivational activity [46]. Moreover, phosphorylation of RUNX3 by Src [47] or PIM1 kinase [48] decreases nuclear import of RUNX3, thereby reducing its transactivation activity. Recently, G9a was shown to interact with RUNX3 [49], suggesting that G9a might directly modulate RUNX3 to control tumor suppressive functions of RUNX3. However, the precise molecular mechanism of RUNX3 methylation by G9a under hypoxia and its links to tumor suppressive functions of RUNX3 were not fully investigated.

In this study, we demonstrated that K129 and K171 are methylated by G9a under hypoxic conditions. The acetylation and methylation at K171 led to activation and inactivation of RUNX3, respectively, under different conditions (e.g., serum-stimulated and hypoxic conditions). Accordingly, these two PTMs at K171 may not occur simultaneously because RUNX3 is likely to be activated or inactivated under specific condition. Although p300, BRD2, and G9a interact with the same Runt domain of RUNX3, these data suggest that cells undergo differential lysine modifications of RUNX3 through interactions with these proteins to adequately adapt them to the varying tumor microenvironment. In particular, tumor cells appear to have methylation of K129 and K171 as the predominant lysine modifications under hypoxic microenvironment.

Regarding the reduced activity of promoter binding to the RUNX3 target genes (Figs. 5 and 6), we can suggest the two possible explanations from the results (Fig. 4), the time-dependent RUNX3 expression pattern, and RUNX3-G9a interaction in the nucleus at 8 h under hypoxia (Fig. S1C–E). One possibility is that the early increase of G9a under hypoxia methylates RUNX3, and the methylation of RUNX3 inhibits the interaction with CBFβ and p300, key cofactors to activate RUNX3 transactivation activity [45, 50]. The decreased interaction of RUNX3 with these cofactors would be an essential mechanism of reduced binding activity to the target gene promoters when RUNX3 is abundant in the nucleus in the primary stage of hypoxic exposure. Further, methylated RUNX3 dissociate with importin-α7 and translocate into the cytosol resulting in the reduced amount of the RUNX3 protein in the nucleus. This methylation-induced cytosolic translocation and degradation would be another possible mechanism to decrease RUNX3 transactivation activity. Most RUNX3 was distributed in the cytoplasm under hypoxia, while a little RUNX3 was localized in the nucleus. RUNX3 methylation by G9a decreased nuclear localization of RUNX3 under hypoxia, which might induce the RUNX3 protein degradation by the cytosolic mislocalization [51], resulting in the reduction of its transactivation activity. Inhibition of nuclear export by leptomycin redistributed RUNX3 in the nucleus under hypoxia (Fig. 2F), which blocked translocation of RUNX3 to the cytoplasm and accumulated RUNX3 in the nucleus without protein degradation. Therefore, G9a induced less nuclear localization of RUNX3 by dissociating of RUNX3 with importin-α7 in the nucleus, but not by inhibiting nuclear import of RUNX3 in the cytoplasm. Therefore, RUNX3 methylation by G9a reduced RUNX3 transactivation activity and increased protein degradation by the cytosolic localization, which finally promoted tumor progression by the reduction of a tumor suppressor.

Hypoxia is highly associated with the early stage of tumorigenesis [52, 53]. We showed that G9a-mediated methylation led to RUNX3 inactivation under hypoxia during early tumorigenesis. Our association analyses revealed that somatic mutations in the neighboring regions of K129 and K171 decreased G9a-mediated methylation of RUNX3, and higher basal levels of RUNX3 at early stage of tumorigenesis showed better patient survival. Accordingly, somatic mutations and high basal levels of RUNX3 can determine the rate of RUNX3 inactivation by G9a-mediated methylation during early tumorigenesis, thereby affecting patient survival. G9a-mediated RUNX3 methylation thus can serve as a potential therapeutic target to prevent tumor growth by increasing cell proliferation and antiapotosis during early tumorigenesis.

## Supplementary information

Figure S1

Figure S2

Figure S3

Figure S4

Figure S5

Table S1

Table S2

Table S3

Table S4

RUNX3-meth-Supplementary-CDD-R
