# Supplementary figures and images for: RUNX3 methylation drives hypoxia-induced cell proliferation and antiapoptosis in early tumorigenesis
Source: Cell Death Differ. 2020 Oct 28;28(4):1251–69. doi: 10.1038/s41418-020-00647-1 (PMC8027031; doi:10.1038/s41418-020-00647-1)

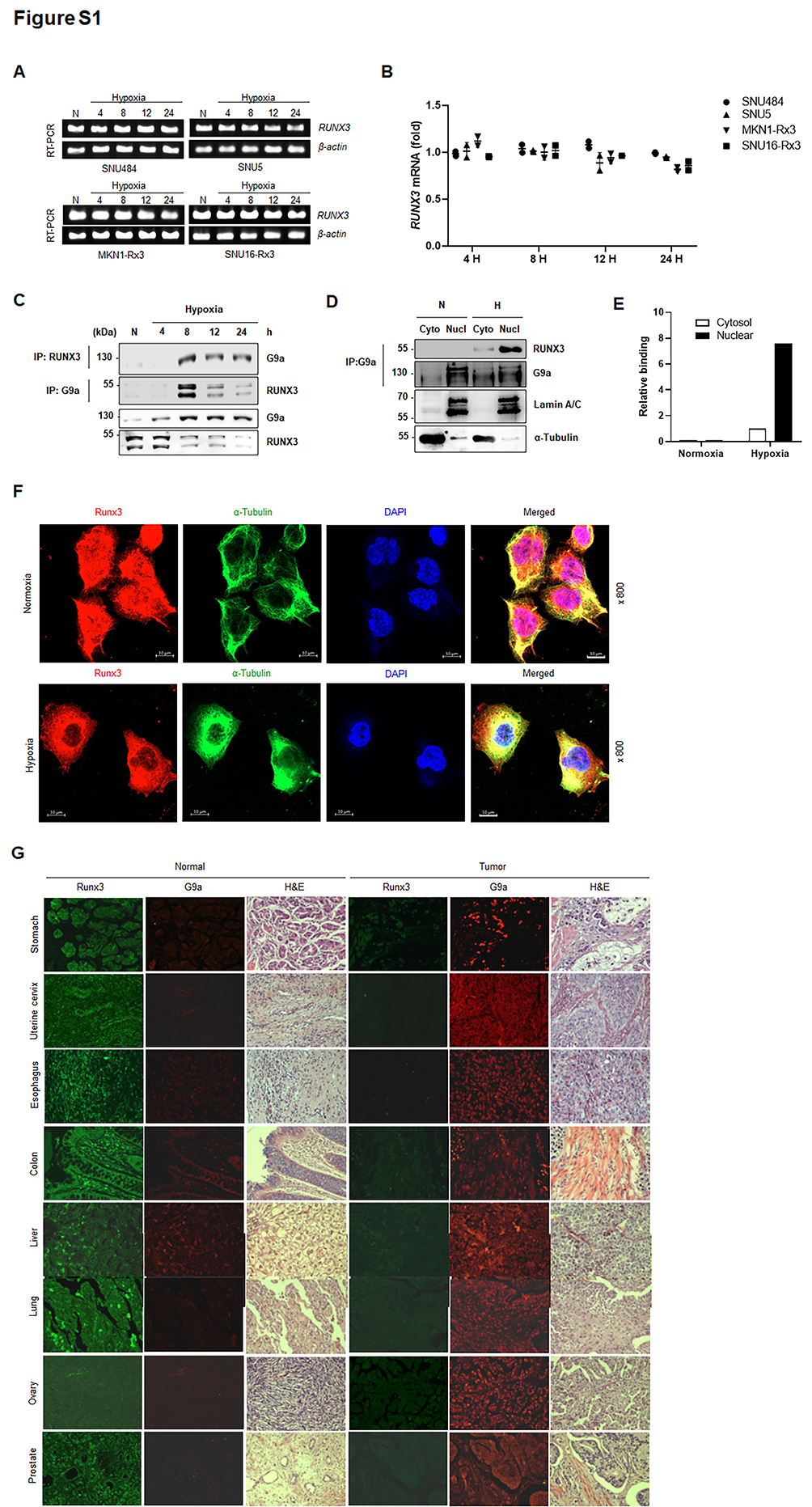

Supplement: Supplementary file 1 — Figure S1 [file 41418_2020_647_MOESM1_ESM.tif]

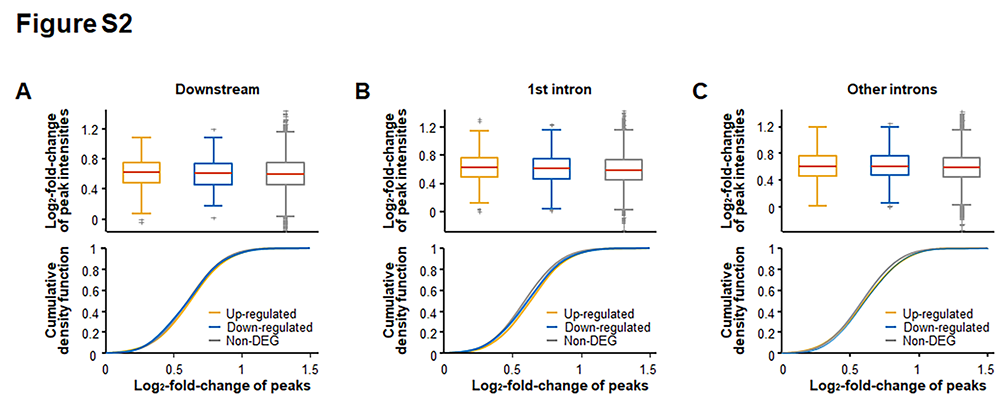

Supplement: Supplementary file 2 — Figure S2 [file 41418_2020_647_MOESM2_ESM.tif]

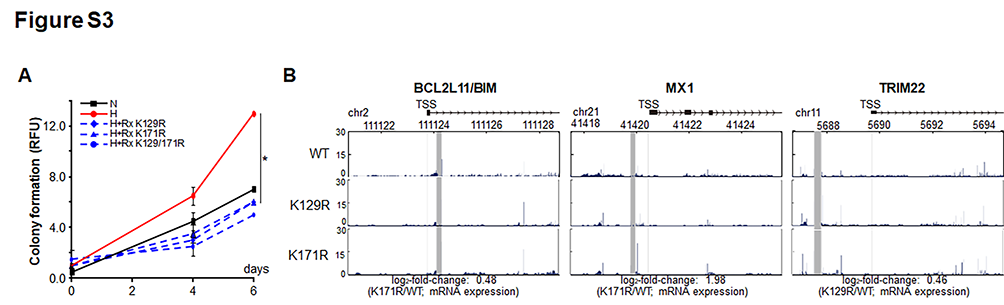

Supplement: Supplementary file 3 — Figure S3 [file 41418_2020_647_MOESM3_ESM.tif]

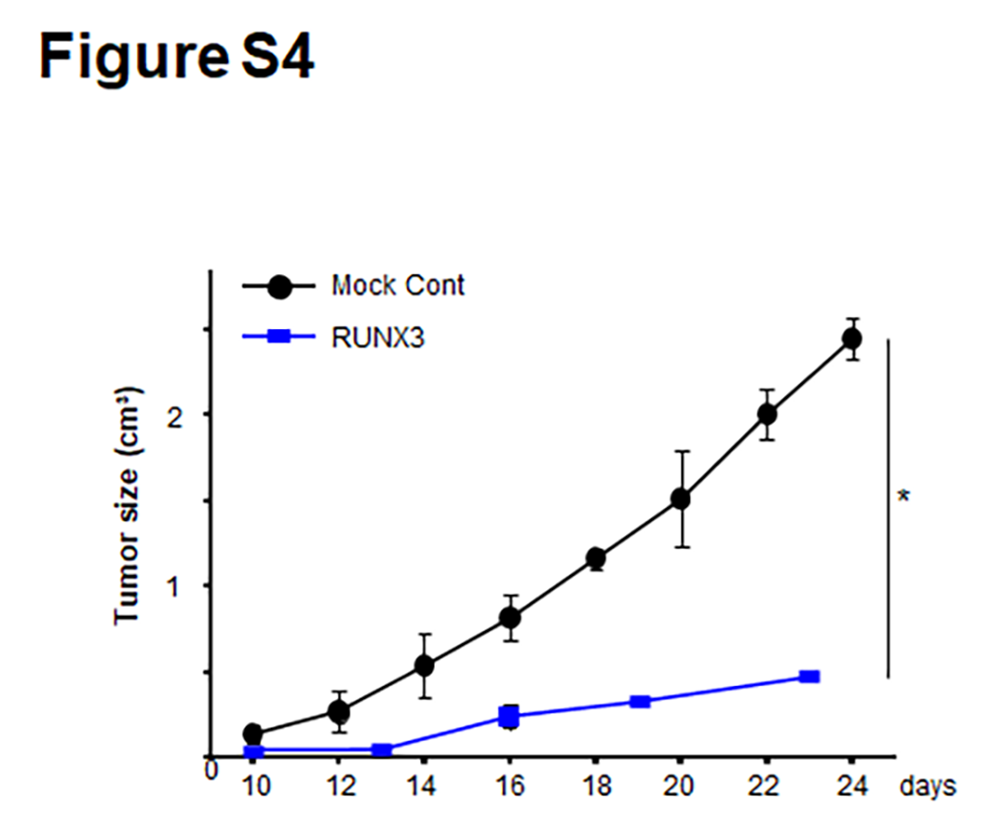

Supplement: Supplementary file 4 — Figure S4 [file 41418_2020_647_MOESM4_ESM.tif]

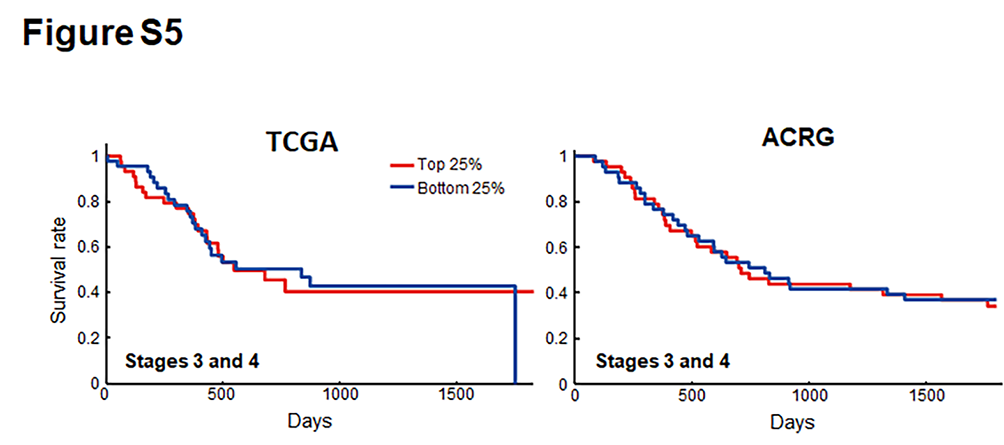

Supplement: Supplementary file 5 — Figure S5 [file 41418_2020_647_MOESM5_ESM.tif]
