# Supplementary material for: RUNX3 methylation drives hypoxia-induced cell proliferation and antiapoptosis in early tumorigenesis
Source: Cell Death Differ. 2020 Oct 28;28(4):1251–69. doi: 10.1038/s41418-020-00647-1 (PMC8027031; doi:10.1038/s41418-020-00647-1)
Supplement: Supplementary file 6 — Table S1 [file 41418_2020_647_MOESM6_ESM.pdf]

Table S1. List of differentially expressed genes by K129R mutation.

Annotation					
Entrez ID	Gene Symbol	Gene Description	UP/DOWN	log2-fold -change	overall p-value
26	AOC1	amine oxidase, copper containing 1	UP	0.768	0.001028041
133	ADM	adrenomedullin	UP	0.61	0.0020027
330	BIRC3	baculoviral IAP repeat containing 3	UP	0.414	0.004762932
379	ARL4D	ADP-ribosylation factor-like 4D	UP	0.421	0.001445451
467	ATF3	activating transcription factor 3	UP	0.469	0.005116034
654	BMP6	bone morphogenetic protein 6	UP	0.408	0.00568445
894	CCND2	cyclin D2	UP	0.428	0.004267061
969	CD69	CD69 molecule	UP	0.483	0.000335913
1044	CDX1	caudal type homeobox 1	UP	0.796	0.001205015
1075	CTSC	cathepsin C	UP	0.509	0.00691458
1264	CNN1	calponin 1, basic, smooth muscle	UP	0.467	0.002851777
1306	COL15A1	collagen, type XV, alpha 1	UP	0.413	0.000549323
1412	CRYBA2	crystallin, beta A2	UP	0.486	0.00236279
1437	CSF2	colony stimulating factor 2 (granulocyte-macrophage)	UP	1.379	9.52762E-05
1440	CSF3	colony stimulating factor 3 (granulocyte)	UP	0.697	0.000466813
1573	CYP2J2	cytochrome P450, family 2, subfamily J, polypeptide 2	UP	0.439	0.001066435
1649	DDIT3	DNA-damage-inducible transcript 3	UP	0.44	0.0024525
1848	DUSP6	dual specificity phosphatase 6	UP	0.754	0.003146517
1870	E2F2	E2F transcription factor 2	UP	0.453	0.000308404
1901	S1PR1	sphingosine-1-phosphate receptor 1	UP	0.573	0.003666134
1959	EGR2	early growth response 2	UP	0.743	0.003014947

2069	EREG	epiregulin	UP	0.532	5.70573E-05
2172	FABP6	fatty acid binding protein 6, ileal	UP	0.415	0.00170683
2201	FBN2	fibrillin 2	UP	0.468	0.000360388
2248	FGF3	fibroblast growth factor 3	UP	0.706	7.25047E-06
2300	FOXL1	forkhead box L1	UP	0.459	0.002742349
2307	FOXS1	forkhead box S1	UP	1.089	0.000212747
2537	IFI6	interferon, alpha-inducible protein 6	UP	2.036	7.50604E-06
2635	GBP3	guanylate binding protein 3	UP	0.485	0.001095685
2706	GJB2	gap junction protein, beta 2, 26kDa	UP	0.429	0.002465599
2854	GPR32	G protein-coupled receptor 32	UP	0.596	0.00147471
2919	CXCL1	chemokine (C-X-C motif) ligand 1<br>(melanoma growth stimulating activity, alpha)	UP	0.465	0.00089749
3002	GZMB	granzyme B<br>(granzyme 2, cytotoxic T-lymphocyte-associated serine esterase 1)	UP	1.628	2.85521E-05
3123	HLA-DRB1	major histocompatibility complex, class II, DR beta 1	UP	0.454	0.004481982
3162	HMOX1	heme oxygenase 1	UP	0.415	0.007555812
3212	HOXB2	homeobox B2	UP	0.479	0.002414697
3242	HPD	4-hydroxyphenylpyruvate dioxygenase	UP	0.47	0.000991029
3310	HSPA6	heat shock 70kDa protein 6 (HSP70B')	UP	1.36	0.000471228
3384	ICAM2	intercellular adhesion molecule 2	UP	0.454	0.001111101
3428	IFI16	interferon, gamma-inducible protein 16	UP	0.411	0.00344826
3429	IFI27	interferon, alpha-inducible protein 27	UP	3.876	3.44573E-07
3430	IFI35	interferon-induced protein 35	UP	0.492	0.001690976
3431	SP110	SP110 nuclear body protein	UP	0.476	0.000696539
3433	IFIT2	interferon-induced protein with tetratricopeptide repeats 2	UP	1.054	0.000375398
3434	IFIT1	interferon-induced protein with tetratricopeptide repeats 1	UP	2.208	1.08753E-05
3437	IFIT3	interferon-induced protein with tetratricopeptide repeats 3	UP	0.861	0.001240444
3549	IHH	indian hedgehog	UP	0.778	0.001819924
3552	IL1A	interleukin 1, alpha	UP	0.751	0.002909969

3553	IL1B	interleukin 1, beta	UP	0.994	9.96486E-05
3569	IL6	interleukin 6	UP	0.902	0.000195949
3576	CXCL8	chemokine (C-X-C motif) ligand 8	UP	0.544	3.13352E-05
3589	IL11	interleukin 11	UP	0.482	0.002401207
3620	IDO1	indoleamine 2,3-dioxygenase 1	UP	0.661	0.000247281
3624	INHBA	inhibin, beta A	UP	0.469	0.003053666
3627	CXCL10	chemokine (C-X-C motif) ligand 10	UP	0.901	0.000375169
3669	ISG20	interferon stimulated exonuclease gene 20kDa	UP	0.784	0.00150242
3690	ITGB3	integrin, beta 3 (platelet glycoprotein IIIa, antigen CD61)	UP	0.748	0.001468318
3834	KIF25	kinesin family member 25	UP	0.451	0.003386307
3885	KRT34	keratin 34, type I	UP	0.708	0.00013431
3914	LAMB3	laminin, beta 3	UP	0.541	0.004414039
3954	LETM1	leucine zipper-EF-hand containing transmembrane protein 1	UP	0.458	0.003960856
3965	LGALS9	lectin, galactoside-binding, soluble, 9	UP	0.411	0.009731514
4188	MDFI	MyoD family inhibitor	UP	0.837	0.003072544
4318	MMP9	matrix metallopeptidase 9	UP	0.589	0.002891779
4501	MT1X	metallothionein 1X	UP	0.485	0.005417241
4599	MX1	MX dynamin-like GTPase 1	UP	1.969	1.31739E-05
4609	MYC	v-myc avian myelocytomatosis viral oncogene homolog	UP	0.554	0.0003679
4843	NOS2	nitric oxide synthase 2, inducible	UP	0.635	0.002041712
4860	PNP	purine nucleoside phosphorylase	UP	0.416	0.000387698
4938	OAS1	2'-5'-oligoadenylate synthetase 1, 40/46kDa	UP	1.285	0.000150234
4939	OAS2	2'-5'-oligoadenylate synthetase 2, 69/71kDa	UP	1.792	7.43072E-06
4940	OAS3	2'-5'-oligoadenylate synthetase 3, 100kDa	UP	1.125	0.00025248
5055	SERPINB2	serpin peptidase inhibitor, clade B (ovalbumin), member 2	UP	0.661	0.004441426
5138	PDE2A	phosphodiesterase 2A, cGMP-stimulated	UP	0.457	0.003340974
5271	SERPINB8	serpin peptidase inhibitor, clade B (ovalbumin), member 8	UP	0.446	0.002011668
5328	PLAU	plasminogen activator, urokinase	UP	0.712	0.000331114
5359	PLSCR1	phospholipid scramblase 1	UP	0.653	0.001027113

5454	POU3F2	POU class 3 homeobox 2	UP	0.415	0.003655641
5743	PTGS2	prostaglandin-endoperoxide synthase 2 (prostaglandin G/H synthase and cyclooxygenase)	UP	0.466	0.00157975
5801	PTPRR	protein tyrosine phosphatase, receptor type, R	UP	0.514	0.000221804
5806	PTX3	pentraxin 3, long	UP	0.427	0.002344354
5997	RGS2	regulator of G-protein signaling 2	UP	0.568	0.004400024
6004	RGS16	regulator of G-protein signaling 16	UP	0.491	0.007130983
6273	S100A2	S100 calcium binding protein A2	UP	0.703	4.20052E-05
6346	CCL1	chemokine (C-C motif) ligand 1	UP	0.918	0.000524979
6348	CCL3	chemokine (C-C motif) ligand 3	UP	1.453	9.13344E-05
6352	CCL5	chemokine (C-C motif) ligand 5	UP	0.538	0.000920721
6591	SNAI2	snail family zinc finger 2	UP	0.61	0.003631756
6616	SNAP25	synaptosomal-associated protein, 25kDa	UP	0.408	0.002198001
6690	SPINK1	serine peptidase inhibitor, Kazal type 1	UP	1.406	2.43028E-05
6696	SPP1	secreted phosphoprotein 1	UP	0.43	0.004402592
6773	STAT2	signal transducer and activator of transcription 2, 113kDa	UP	0.554	0.000371332
6915	TBXA2R	thromboxane A2 receptor	UP	0.424	0.003244945
7098	TLR3	toll-like receptor 3	UP	0.421	0.006390288
7128	TNFAIP3	tumor necrosis factor, alpha-induced protein 3	UP	0.742	0.002382995
7130	TNFAIP6	tumor necrosis factor, alpha-induced protein 6	UP	0.478	0.002302539
7185	TRAF1	TNF receptor-associated factor 1	UP	0.757	0.001459593
7378	UPP1	uridine phosphorylase 1	UP	0.793	0.000880817
7421	VDR	vitamin D (1,25- dihydroxyvitamin D3) receptor	UP	0.65	0.001227401
8013	NR4A3	nuclear receptor subfamily 4, group A, member 3	UP	0.509	0.003283564
8091	HMGA2	high mobility group AT-hook 2	UP	0.682	0.001207026
8241	RBM10	RNA binding motif protein 10	UP	0.419	0.007472288
8309	ACOX2	acyl-CoA oxidase 2, branched chain	UP	0.582	0.000170035
8482	SEMA7A	semaphorin 7A, GPI membrane anchor (John Milton Hagen blood group)	UP	0.494	0.000439693

8519	IFITM1	interferon induced transmembrane protein 1	UP	0.902	0.000823787
8553	BHLHE40	basic helix-loop-helix family, member e40	UP	0.456	0.001973811
8638	OASL	2'-5'-oligoadenylate synthetase-like	UP	1.219	0.000268171
8644	AKR1C3	aldo-keto reductase family 1, member C3	UP	0.419	0.003968272
8728	ADAM19	ADAM metallopeptidase domain 19	UP	0.429	0.00217094
8739	HRK	harakiri, BCL2 interacting protein	UP	0.426	0.004463927
8743	TNFSF10	tumor necrosis factor (ligand) superfamily, member 10	UP	0.722	0.001075674
9023	CH25H	cholesterol 25-hydroxylase	UP	0.913	0.000257464
9120	SLC16A6	solute carrier family 16, member 6	UP	0.685	0.003400979
9241	NOG	noggin	UP	0.41	0.007647553
9423	NTN1	netrin 1	UP	0.564	0.005070413
9518	GDF15	growth differentiation factor 15	UP	0.89	0.002257224
9636	ISG15	ISG15 ubiquitin-like modifier	UP	1.292	0.000238789
9729	KIAA0408	KIAA0408	UP	0.451	0.004923391
9935	MAFB	v-maf avian musculoaponeurotic fibrosarcoma oncogene homolog B	UP	1.108	0.000453086
9985	REC8	REC8 meiotic recombination protein	UP	0.539	0.001941109
10344	CCL26	chemokine (C-C motif) ligand 26	UP	0.504	6.9363E-05
10346	TRIM22	tripartite motif containing 22	UP	0.458	0.007488809
10379	IRF9	interferon regulatory factor 9	UP	1.695	1.94334E-05
10544	PROCR	protein C receptor, endothelial	UP	0.51	0.000528648
10561	IFI44	interferon-induced protein 44	UP	1.353	3.78289E-05
10961	ERP29	endoplasmic reticulum protein 29	UP	0.578	0.002135974
10964	IFI44L	interferon-induced protein 44-like	UP	2.321	1.53839E-05
11095	ADAMTS8	ADAM metallopeptidase with thrombospondin type 1 motif, 8	UP	0.482	0.004440469
11097	NUPL2	nucleoporin like 2	UP	0.481	0.00270217
11274	USP18	ubiquitin specific peptidase 18	UP	0.646	0.001794635
23331	TTC28	tetratricopeptide repeat domain 28	UP	0.427	0.007239629
23553	HYAL4	hyaluronoglucosaminidase 4	UP	0.416	0.00860049
23586	DDX58	DEAD (Asp-Glu-Ala-Asp) box polypeptide 58	UP	0.833	0.000852291

25895	METTL21B	methyltransferase like 21B	UP	0.47	0.00587759
26150	RIBC2	RIB43A domain with coiled-coils 2	UP	0.426	0.001151587
26585	GREM1	gremlin 1, DAN family BMP antagonist	UP	0.532	0.000531696
26872	STEAP1	six transmembrane epithelial antigen of the prostate 1	UP	0.672	0.000800933
27033	ZBTB32	zinc finger and BTB domain containing 32	UP	0.715	0.004250901
27074	LAMP3	lysosomal-associated membrane protein 3	UP	0.476	0.00211008
27290	SPINK4	serine peptidase inhibitor, Kazal type 4	UP	0.417	0.007875678
29780	PARVB	parvin, beta	UP	0.976	0.001025794
50964	SOST	sclerostin	UP	0.798	6.27435E-05
51176	LEF1	lymphoid enhancer-binding factor 1	UP	0.637	0.000653959
51296	SLC15A3	solute carrier family 15 (oligopeptide transporter), member 3	UP	0.478	0.006182953
51299	NRN1	neuritin 1	UP	0.435	0.006208986
51561	IL23A	interleukin 23, alpha subunit p19	UP	1.02	0.000420099
51655	RASD1	RAS, dexamethasone-induced 1	UP	0.589	9.20645E-05
51676	ASB2	ankyrin repeat and SOCS box containing 2	UP	0.429	0.006832253
54210	TREM1	triggering receptor expressed on myeloid cells 1	UP	0.544	0.001034962
54538	ROBO4	roundabout guidance receptor 4	UP	0.489	0.006809472
54625	PARP14	poly (ADP-ribose) polymerase family, member 14	UP	0.52	0.003178996
54739	XAF1	XIAP associated factor 1	UP	1.054	0.000725452
54742	LY6K	lymphocyte antigen 6 complex, locus K	UP	0.445	0.001874391
54754	NUTM2F	NUT family member 2F	UP	0.443	0.007180657
54993	ZSCAN2	zinc finger and SCAN domain containing 2	UP	0.488	0.004003677
55007	FAM118A	family with sequence similarity 118, member A	UP	0.481	0.0020198
55076	TMEM45A	transmembrane protein 45A	UP	0.453	0.000517574
55601	DDX60	DEAD (Asp-Glu-Ala-Asp) box polypeptide 60	UP	0.753	0.00246881
55733	HHAT	hedgehog acyltransferase	UP	0.588	0.001167585
55790	CSGALNACT1	chondroitin sulfate N-acetylgalactosaminyltransferase 1	UP	0.47	0.004205968
56300	IL36G	interleukin 36, gamma	UP	0.723	0.001069222
56829	ZC3HAV1	zinc finger CCCH-type, antiviral 1	UP	0.463	0.001448499

57608	KIAA1462	KIAA1462	UP	0.492	0.000315979
57692	MAGEE1	melanoma antigen family E1	UP	0.42	0.001022662
57801	HES4	hes family bHLH transcription factor 4	UP	0.456	0.00547847
64108	RTP4	receptor (chemosensory) transporter protein 4	UP	0.416	0.00214039
64135	IFIH1	interferon induced with helicase C domain 1	UP	0.735	0.00105749
79160	LOC79160	uncharacterized LOC79160	UP	0.431	0.002823253
79413	ZBED2	zinc finger, BED-type containing 2	UP	0.729	0.003638553
79695	GALNT12	polypeptide N-acetylgalactosaminyltransferase 12	UP	0.501	0.006375585
80896	NPL	N-acetylneuraminate pyruvate lyase (dihydrodipicolinate synthase)	UP	0.409	0.002018923
83538	TTC25	tetratricopeptide repeat domain 25	UP	0.468	0.002432345
83666	PARP9	poly (ADP-ribose) polymerase family, member 9	UP	0.641	0.003161238
83850	ESYT3	extended synaptotagmin-like protein 3	UP	0.472	0.0023248
84419	C15orf48	chromosome 15 open reading frame 48	UP	0.544	0.002353014
84717	HDGFRP2	hepatoma-derived growth factor-related protein 2	UP	0.467	0.000174316
84879	MFSD2A	major facilitator superfamily domain containing 2A	UP	0.503	0.002741712
84951	TNS4	tensin 4	UP	0.686	0.00182232
85441	HELZ2	helicase with zinc finger 2, transcriptional coactivator	UP	0.43	0.006095733
85463	ZC3H12C	zinc finger CCCH-type containing 12C	UP	0.47	0.002261343
91543	RSAD2	radical S-adenosyl methionine domain containing 2	UP	2.216	2.7641E-05
93429	DKFZp434J0226	uncharacterized LOC93429	UP	0.467	0.004301547
94240	EPSTI1	epithelial stromal interaction 1 (breast)	UP	0.604	0.000395563
114548	NLRP3	NLR family, pyrin domain containing 3	UP	0.48	0.003027838
114801	TMEM200A	transmembrane protein 200A	UP	0.562	0.000396168
114907	FBXO32	F-box protein 32	UP	0.68	0.001164482
115123	MARCH3	membrane-associated ring finger (C3HC4) 3, E3 ubiquitin protein ligase	UP	0.434	0.001510731
115265	DDIT4L	DNA-damage-inducible transcript 4-like	UP	0.628	0.002327573
116071	BATF2	basic leucine zipper transcription factor, ATF-like 2	UP	0.494	0.006807265

125965	COX6B2	cytochrome c oxidase subunit VIb polypeptide 2 (testis)	UP	0.418	0.006569953
129607	CMPK2	cytidine monophosphate (UMP-CMP) kinase 2, mitochondrial	UP	0.744	7.21636E-05
130827	TMEM182	transmembrane protein 182	UP	0.42	0.008566365
140686	WFDC3	WAP four-disulfide core domain 3	UP	0.716	0.001471883
145200	LINC00239	long intergenic non-protein coding RNA 239	UP	0.441	0.006366712
146512	FLJ30679	uncharacterized protein FLJ30679	UP	0.414	0.007319213
149773	APCDD1L-AS1	APCDD1L antisense RNA 1 (head to head)	UP	0.65	0.000754522
152816	C4orf26	chromosome 4 open reading frame 26	UP	0.828	0.000702412
158511	CSAG1	chondrosarcoma associated gene 1	UP	0.822	0.000344057
160428	ALDH1L2	aldehyde dehydrogenase 1 family, member L2	UP	0.561	0.002064053
163351	GBP6	guanylate binding protein family, member 6	UP	0.427	0.005379304
203413	CT83	cancer/testis antigen 83	UP	0.564	0.003376802
203430	ZCCHC5	zinc finger, CCHC domain containing 5	UP	0.418	0.00570981
219285	SAMD9L	sterile alpha motif domain containing 9-like	UP	0.733	0.000467386
219699	UNC5B	unc-5 netrin receptor B	UP	0.54	0.004748207
219874	OR6T1	olfactory receptor, family 6, subfamily T, member 1	UP	0.464	0.004252644
246721	POLR2J2	polymerase (RNA) II (DNA directed) polypeptide J2	UP	0.503	0.001234567
256227	STEAP1B	STEAP family member 1B	UP	0.722	0.000851364
256949	KANK3	KN motif and ankyrin repeat domains 3	UP	0.478	0.005094488
256979	SUN3	Sad1 and UNC84 domain containing 3	UP	0.499	0.003002701
282616	IFNL2	interferon, lambda 2	UP	0.677	0.002986159
283335	LOC283335	uncharacterized LOC283335	UP	0.481	0.003559792
284276	LINC00908	long intergenic non-protein coding RNA 908	UP	0.525	0.000713917
284454	LOC284454	uncharacterized LOC284454	UP	0.482	0.004066627
340037	PRR7-AS1	PRR7 antisense RNA 1	UP	0.423	0.001952307
340390	WDR97	WD repeat domain 97	UP	0.513	0.00223875
349075	ZNF713	zinc finger protein 713	UP	0.453	0.006545729
374383	NCR3LG1	natural killer cell cytotoxicity receptor 3 ligand 1	UP	0.429	0.002608275
374897	SBSN	suprabasin	UP	0.471	0.001421998

388394	RPRML	reprimo-like	UP	0.599	0.001571807
389332	LOC389332	uncharacterized LOC389332	UP	0.823	0.001908003
390748	PABPN1L	poly(A) binding protein, nuclear 1-like (cytoplasmic)	UP	0.841	0.002325318
400745	SH2D5	SH2 domain containing 5	UP	0.459	0.00513172
401647	GOLGA7B	golgin A7 family, member B	UP	0.531	0.001190887
642691	FLJ37786	uncharacterized LOC642691	UP	0.691	0.003915916
654346	LGALS9C	lectin, galactoside-binding, soluble, 9C	UP	0.557	0.003788354
728461	#N/A	#N/A	UP	0.64	0.002808461
100130476	LOC100130476	uncharacterized LOC100130476	UP	0.448	0.006732963
100130935	CSAG4	CSAG family, member 4 (pseudogene)	UP	0.81	0.000612118
100130938	#N/A	#N/A	UP	0.738	0.000928803
100131390	SP9	Sp9 transcription factor	UP	0.507	0.000720516
100216001	LINC00704	long intergenic non-protein coding RNA 704	UP	0.843	0.000376314
100505702	LINC01094	long intergenic non-protein coding RNA 1094	UP	1.287	0.000118702
100506178	LOC100506178	uncharacterized LOC100506178	UP	0.641	0.002737058
100506190	LINC00963	long intergenic non-protein coding RNA 963	UP	0.46	0.003972617
100506377	LINC00973	long intergenic non-protein coding RNA 973	UP	0.771	0.001122827
100506718	LOC100506718	uncharacterized LOC100506718	UP	0.46	0.001646243
100507144	LOC100507144	uncharacterized LOC100507144	UP	0.486	0.001896743
100507410	C1QTNF1-AS1	C1QTNF1 antisense RNA 1	UP	0.576	0.00295192
100509445	#N/A	#N/A	UP	0.489	0.002744282
100652988	LINC00702	long intergenic non-protein coding RNA 702	UP	0.55	0.004183216
100996267	#N/A	#N/A	UP	1.105	0.002016093
101928841	LOC101928841	collagen alpha-1(II) chain-like	UP	0.62	0.002304427
101929585	#N/A	#N/A	UP	0.424	0.000350235
101930294	LOC101930294	uncharacterized LOC101930294	UP	0.78	0.001921258
250	ALPP	alkaline phosphatase, placental	DOWN	-0.88	0.000646036
444	ASPH	aspartate beta-hydroxylase	DOWN	-0.643	0.000828757
827	CAPN6	calpain 6	DOWN	-0.439	0.006677618

1295	COL8A1	collagen, type VIII, alpha 1	DOWN	-0.62	0.001381065
1495	CTNNA1	catenin (cadherin-associated protein), alpha 1, 102kDa	DOWN	-0.407	0.006318593
1917	EEF1A2	eukaryotic translation elongation factor 1 alpha 2	DOWN	-0.426	0.004338873
2969	GTF2I	general transcription factor IIi	DOWN	-0.439	0.001159195
3024	HIST1H1A	histone cluster 1, H1a	DOWN	-0.44	0.004128402
3149	HMGB3	high mobility group box 3	DOWN	-0.437	0.00924324
3397	ID1	inhibitor of DNA binding 1,<br>dominant negative helix-loop-helix protein	DOWN	-0.506	0.001566792
3455	IFNAR2	interferon (alpha, beta and omega) receptor 2	DOWN	-0.452	0.009056933
3851	KRT4	keratin 4, type II	DOWN	-0.507	0.007429472
3993	LLGL2	lethal giant larvae homolog 2 (Drosophila)	DOWN	-0.548	0.002325341
4046	LSP1	lymphocyte-specific protein 1	DOWN	-0.782	0.000184883
4508	ATP6	ATP synthase F0 subunit 6	DOWN	-0.436	0.002771466
4509	ATP8	ATP synthase F0 subunit 8	DOWN	-0.669	0.000549944
4514	COX3	cytochrome c oxidase III	DOWN	-0.478	0.00708174
4519	CYTB	cytochrome b	DOWN	-0.477	0.001882745
4904	YBX1	Y box binding protein 1	DOWN	-0.496	0.007268536
5089	PBX2	pre-B-cell leukemia homeobox 2	DOWN	-0.455	0.003724546
5174	PDZK1	PDZ domain containing 1	DOWN	-0.533	0.005890334
5343	PLGLB1	plasminogen-like B1	DOWN	-0.426	0.007122561
5624	PROC	protein C (inactivator of coagulation factors Va and VIIIa)	DOWN	-0.448	0.004306884
5650	KLK7	kallikrein-related peptidase 7	DOWN	-0.656	0.001764203
5653	KLK6	kallikrein-related peptidase 6	DOWN	-0.482	0.001783694
5918	RARRES1	retinoic acid receptor responder (tazarotene induced) 1	DOWN	-0.639	0.004937081
6157	RPL27A	ribosomal protein L27a	DOWN	-0.414	0.008865671
6751	SSTR1	somatostatin receptor 1	DOWN	-0.41	0.000301099
7010	TEK	TEK tyrosine kinase, endothelial	DOWN	-0.474	0.008352736
7049	TGFBR3	transforming growth factor, beta receptor III	DOWN	-0.533	0.00247152
7123	CLEC3B	C-type lectin domain family 3, member B	DOWN	-0.566	0.00511814

7134	TNNC1	troponin C type 1 (slow)	DOWN	-0.541	0.00438061
7266	DNAJC7	DnaJ (Hsp40) homolog, subfamily C, member 7	DOWN	-0.503	0.007801606
7748	ZNF195	zinc finger protein 195	DOWN	-0.435	0.001269997
8148	TAF15	TAF15 RNA polymerase II,<br>TATA box binding protein (TBP)-associated factor, 68kDa	DOWN	-0.53	0.001458645
8581	LY6D	lymphocyte antigen 6 complex, locus D	DOWN	-0.452	0.008801844
8786	RGS11	regulator of G-protein signaling 11	DOWN	-0.418	0.002400812
8817	FGF18	fibroblast growth factor 18	DOWN	-0.416	0.00367665
9204	ZMYM6	zinc finger, MYM-type 6	DOWN	-0.43	0.00519237
9295	SRSF11	serine/arginine-rich splicing factor 11	DOWN	-0.486	0.002617747
9615	GDA	guanine deaminase	DOWN	-0.407	0.001393405
9750	FAM65B	family with sequence similarity 65, member B	DOWN	-0.412	0.000826363
9905	SGSM2	small G protein signaling modulator 2	DOWN	-0.471	0.0003035
9980	DOPEY2	dopey family member 2	DOWN	-0.418	0.003193494
9982	FGFBP1	fibroblast growth factor binding protein 1	DOWN	-0.503	0.001392465
10207	INADL	InaD-like (Drosophila)	DOWN	-0.457	0.001926334
10432	RBM14	RNA binding motif protein 14	DOWN	-0.54	0.000271537
10446	LRRN2	leucine rich repeat neuronal 2	DOWN	-0.432	0.003838695
10451	VAV3	vav 3 guanine nucleotide exchange factor	DOWN	-0.517	0.003449656
10625	IVNS1ABP	influenza virus NS1A binding protein	DOWN	-0.43	0.002907817
10801	SEPT9	septin 9	DOWN	-0.474	0.005077369
11037	STON1	stonin 1	DOWN	-0.47	0.000324131
11076	TPPP	tubulin polymerization promoting protein	DOWN	-0.691	0.002821284
23126	POGZ	pogo transposable element with ZNF domain	DOWN	-0.409	0.002219041
23244	PDS5A	PDS5 cohesin associated factor A	DOWN	-0.414	0.004356321
25929	GEMIN5	gem (nuclear organelle) associated protein 5	DOWN	-0.485	0.002155776
26793	SNORD56	small nucleolar RNA, C/D box 56	DOWN	-0.436	0.001917072
29053	PRO0628	uncharacterized LOC29053	DOWN	-0.43	0.005455709
50808	AK3	adenylate kinase 3	DOWN	-0.407	0.003701958

51593	SRRT	serrate, RNA effector molecule	DOWN	-0.598	0.001722417
53405	CLIC5	chloride intracellular channel 5	DOWN	-0.453	0.003506028
54674	LRRN3	leucine rich repeat neuronal 3	DOWN	-0.82	0.001001504
54873	PALMD	palmdelphin	DOWN	-0.497	0.00073456
54919	DNAAF5	dynein, axonemal, assembly factor 5	DOWN	-0.508	0.00742571
55547	HAB1	B1 for mucin	DOWN	-0.415	0.005653121
55607	PPP1R9A	protein phosphatase 1, regulatory subunit 9A	DOWN	-0.422	0.001902211
55795	PCID2	PCI domain containing 2	DOWN	-0.422	0.001374929
55806	HR	hair growth associated	DOWN	-0.563	0.000237404
56241	SUSD2	sushi domain containing 2	DOWN	-0.416	0.007300454
57459	GATAD2B	GATA zinc finger domain containing 2B	DOWN	-0.599	0.00013599
57701	NCKAP5L	NCK-associated protein 5-like	DOWN	-0.445	0.008325481
64781	CERK	ceramide kinase	DOWN	-0.573	0.006246045
64925	CCDC71	coiled-coil domain containing 71	DOWN	-0.867	0.002275076
79937	CNTNAP3	contactin associated protein-like 3	DOWN	-0.607	0.000385321
80063	ATF7IP2	activating transcription factor 7 interacting protein 2	DOWN	-0.455	0.004118372
80761	UPK3B	uroplakin 3B	DOWN	-0.451	0.009319921
81794	ADAMTS10	ADAM metallopeptidase with thrombospondin type 1 motif, 10	DOWN	-0.434	0.004758478
83849	SYT15	synaptotagmin XV	DOWN	-0.427	0.005228773
84159	ARID5B	AT rich interactive domain 5B (MRF1-like)	DOWN	-0.516	0.004741526
84734	FAM167B	family with sequence similarity 167, member B	DOWN	-0.432	0.000845163
89846	FGD3	FYVE, RhoGEF and PH domain containing 3	DOWN	-0.456	0.004013998
90133	KRT8P12	keratin 8 pseudogene 12	DOWN	-0.465	0.00889447
92154	MTSS1L	metastasis suppressor 1-like	DOWN	-0.452	0.003401953
93010	B3GNT7	UDP-GlcNAc:betaGal beta-1,3-N-acetylglucosaminyltransferase 7	DOWN	-0.448	0.00441562
113146	AHNAK2	AHNAK nucleoprotein 2	DOWN	-0.47	0.002345079
114814	GNRHR2	gonadotropin-releasing hormone (type 2) receptor 2, pseudogene	DOWN	-0.494	0.002727821
123169	LEO1	LEO1 homolog, Paf1/RNA polymerase II complex component	DOWN	-0.645	0.001814951
128710	SLX4IP	SLX4 interacting protein	DOWN	-0.468	0.001367576

140876	FAM65C	family with sequence similarity 65, member C	DOWN	-0.42	0.002567531
142937	LOC142937	uncharacterized protein BC008131	DOWN	-0.634	0.000328809
146212	KCTD19	potassium channel tetramerization domain containing 19	DOWN	-0.557	0.003095125
154043	CNKSR3	CNKSR family member 3	DOWN	-0.626	0.002735202
157869	SBSPON	somatomedin B and thrombospondin, type 1 domain containing	DOWN	-0.569	0.006225705
163782	KANK4	KN motif and ankyrin repeat domains 4	DOWN	-0.415	0.009712281
164633	CABP7	calcium binding protein 7	DOWN	-0.483	0.005482645
165186	FAM179A	family with sequence similarity 179, member A	DOWN	-0.599	0.002724177
222161	DKFZP586I1420	uncharacterized protein DKFZp586I1420	DOWN	-0.438	0.003647294
283298	OLFML1	olfactomedin-like 1	DOWN	-0.424	0.000231478
283932	FBXL19-AS1	FBXL19 antisense RNA 1 (head to head)	DOWN	-0.496	0.006655905
284219	LOC284219	uncharacterized LOC284219	DOWN	-0.47	0.005960989
285489	DOK7	docking protein 7	DOWN	-0.54	0.006246366
286676	ILDR1	immunoglobulin-like domain containing receptor 1	DOWN	-0.459	0.003021674
339766	MROH2A	maestro heat-like repeat family member 2A	DOWN	-0.674	0.002134495
340335	LOC340335	uncharacterized LOC340335	DOWN	-0.455	0.002694711
342979	PALM3	paralemmin 3	DOWN	-0.457	0.003175816
352954	GATS	GATS, stromal antigen 3 opposite strand	DOWN	-0.452	0.000607624
378938	MALAT1	metastasis associated lung adenocarcinoma transcript 1 (non-protein coding)	DOWN	-0.46	0.006002001
389792	IER5L	immediate early response 5-like	DOWN	-0.606	0.005848291
401261	LOC401261	zinc finger protein ZIC 5	DOWN	-0.568	0.004047217
414777	HCG18	HLA complex group 18 (non-protein coding)	DOWN	-0.45	0.003204752
440910	LOC440910	uncharacterized LOC440910	DOWN	-0.462	0.000217897
441273	SPDYE2	speedy/RINGO cell cycle regulator family member E2	DOWN	-0.46	0.00377776
441490	FTH1P18	ferritin, heavy polypeptide 1 pseudogene 18	DOWN	-0.423	0.002194096
646396	REREP3	arginine-glutamic acid dipeptide (RE) repeats pseudogene 3	DOWN	-0.44	0.008246834
677679	SCARNA3	small Cajal body-specific RNA 3	DOWN	-0.431	0.002296701
728577	CNTNAP3B	contactin associated protein-like 3B	DOWN	-0.55	0.001383089

730183	LOC730183	uncharacterized LOC730183	DOWN	-0.414	0.004771036
100130557	NFYC-AS1	NFYC antisense RNA 1	DOWN	-0.445	0.00266181
100131094	DPP9-AS1	DPP9 antisense RNA 1	DOWN	-0.438	0.008895744
100216546	LINC01004	long intergenic non-protein coding RNA 1004	DOWN	-0.574	0.004372371
100505648	RAD51-AS1	RAD51 antisense RNA 1 (head to head)	DOWN	-0.424	0.001676578
