# Supplementary material for: RUNX3 methylation drives hypoxia-induced cell proliferation and antiapoptosis in early tumorigenesis
Source: Cell Death Differ. 2020 Oct 28;28(4):1251–69. doi: 10.1038/s41418-020-00647-1 (PMC8027031; doi:10.1038/s41418-020-00647-1)
Supplement: Supplementary file 7 — Table S2 [file 41418_2020_647_MOESM7_ESM.pdf]

Table S2. List of differentially expressed genes by K171R mutation.

Annotation					
Entrez ID	Gene Symbol	Gene Description	UP/DOWN	log2-fold -change	overall p-value
16	AARS	alanyl-tRNA synthetase	UP	0.52	0.007240256
26	AOC1	amine oxidase, copper containing 1	UP	0.971	0.001336474
133	ADM	adrenomedullin	UP	0.557	0.00525447
379	ARL4D	ADP-ribosylation factor-like 4D	UP	0.438	0.001740471
467	ATF3	activating transcription factor 3	UP	0.413	0.00023899
595	CCND1	cyclin D1	UP	0.44	0.002386672
631	BFSP1	beaded filament structural protein 1, filensin	UP	0.42	0.001768688
684	BST2	bone marrow stromal cell antigen 2	UP	0.431	0.007371546
969	CD69	CD69 molecule	UP	0.465	0.000374276
1044	CDX1	caudal type homeobox 1	UP	0.87	0.000165766
1050	CEBPA	CCAAT/enhancer binding protein (C/EBP), alpha	UP	0.545	0.001193566
1051	CEBPB	CCAAT/enhancer binding protein (C/EBP), beta	UP	0.528	0.000303844
1075	CTSC	cathepsin C	UP	0.556	0.000753035
1113	CHGA	chromogranin A	UP	0.425	0.004044512
1236	CCR7	chemokine (C-C motif) receptor 7	UP	0.459	0.008505899
1264	CNN1	calponin 1, basic, smooth muscle	UP	0.511	0.003062022
1437	CSF2	colony stimulating factor 2 (granulocyte-macrophage)	UP	1.51	3.18311E-05
1440	CSF3	colony stimulating factor 3 (granulocyte)	UP	0.816	0.002491952
1466	CSRP2	cysteine and glycine-rich protein 2	UP	0.449	0.001958645
1475	CSTA	cystatin A (stefin A)	UP	0.424	0.00183406
1649	DDIT3	DNA-damage-inducible transcript 3	UP	0.603	0.001193016
1839	HBEGF	heparin-binding EGF-like growth factor	UP	0.532	0.008600904

1844	DUSP2	dual specificity phosphatase 2	UP	0.507	0.004352713
1848	DUSP6	dual specificity phosphatase 6	UP	0.68	0.006194461
1870	E2F2	E2F transcription factor 2	UP	0.489	0.001042089
1959	EGR2	early growth response 2	UP	0.754	0.004849697
2014	EMP3	epithelial membrane protein 3	UP	0.425	0.002300568
2118	ETV4	ets variant 4	UP	0.776	0.000703767
2120	ETV6	ets variant 6	UP	0.622	0.005082635
2172	FABP6	fatty acid binding protein 6, ileal	UP	0.523	0.000190303
2248	FGF3	fibroblast growth factor 3	UP	0.734	0.00026212
2260	FGFR1	fibroblast growth factor receptor 1	UP	0.483	0.006073556
2274	FHL2	four and a half LIM domains 2	UP	0.475	0.001133918
2300	FOXL1	forkhead box L1	UP	0.56	0.002513198
2307	FOXS1	forkhead box S1	UP	1.269	0.000482765
2312	FLG	filaggrin	UP	0.66	0.005072562
2318	FLNC	filamin C, gamma	UP	0.702	0.001570173
2335	FN1	fibronectin 1	UP	0.574	0.007379836
2354	FOSB	FBJ murine osteosarcoma viral oncogene homolog B	UP	0.495	0.002165512
2357	FPR1	formyl peptide receptor 1	UP	0.675	0.006358582
2537	IFI6	interferon, alpha-inducible protein 6	UP	2.157	4.16424E-06
2643	GCH1	GTP cyclohydrolase 1	UP	0.528	0.005509084
2706	GJB2	gap junction protein, beta 2, 26kDa	UP	0.582	0.001777041
2709	GJB5	gap junction protein, beta 5, 31.1kDa	UP	0.424	0.001972179
2854	GPR32	G protein-coupled receptor 32	UP	0.619	0.003416537
2912	GRM2	glutamate receptor, metabotropic 2	UP	0.548	0.007902891
2919	CXCL1	chemokine (C-X-C motif) ligand 1 (melanoma growth stimulating activity, alpha)	UP	0.442	0.004269641
2921	CXCL3	chemokine (C-X-C motif) ligand 3	UP	0.424	0.00033951
2990	GUSB	glucuronidase, beta	UP	0.457	0.005699306
3002	GZMB	granzyme B (granzyme 2, cytotoxic T-lymphocyte-associated serine esterase 1)	UP	1.804	1.87201E-05

3106	HLA-B	major histocompatibility complex, class I, B	UP	0.449	0.004384123
3109	HLA-DMB	major histocompatibility complex, class II, DM beta	UP	0.524	0.001520132
3123	HLA-DRB1	major histocompatibility complex, class II, DR beta 1	UP	0.582	0.000237746
3176	HNMT	histamine N-methyltransferase	UP	0.409	0.005480881
3212	HOXB2	homeobox B2	UP	0.575	0.002190772
3241	HPCAL1	hippocalcin-like 1	UP	0.442	0.003035937
3280	HES1	hes family bHLH transcription factor 1	UP	0.421	0.005496592
3310	HSPA6	heat shock 70kDa protein 6 (HSP70B')	UP	1.478	4.61609E-05
3384	ICAM2	intercellular adhesion molecule 2	UP	0.679	0.001308681
3429	IFI27	interferon, alpha-inducible protein 27	UP	3.781	1.28289E-07
3430	IFI35	interferon-induced protein 35	UP	0.69	0.000115485
3431	SP110	SP110 nuclear body protein	UP	0.526	0.001718274
3433	IFIT2	interferon-induced protein with tetratricopeptide repeats 2	UP	0.957	0.000818283
3434	IFIT1	interferon-induced protein with tetratricopeptide repeats 1	UP	2.073	2.986E-05
3437	IFIT3	interferon-induced protein with tetratricopeptide repeats 3	UP	0.892	0.00013224
3549	IHH	indian hedgehog	UP	1.064	0.00010902
3552	IL1A	interleukin 1, alpha	UP	0.937	0.000339825
3553	IL1B	interleukin 1, beta	UP	1.07	8.39665E-05
3563	IL3RA	interleukin 3 receptor, alpha (low affinity)	UP	0.588	0.001826766
3569	IL6	interleukin 6	UP	0.815	0.000374771
3589	IL11	interleukin 11	UP	0.653	0.002378245
3620	IDO1	indoleamine 2,3-dioxygenase 1	UP	0.664	6.3964E-05
3627	CXCL10	chemokine (C-X-C motif) ligand 10	UP	0.912	0.000575264
3665	IRF7	interferon regulatory factor 7	UP	0.616	0.007063414
3669	ISG20	interferon stimulated exonuclease gene 20kDa	UP	0.892	6.92662E-05
3678	ITGA5	integrin, alpha 5 (fibronectin receptor, alpha polypeptide)	UP	0.461	0.005369868
3690	ITGB3	integrin, beta 3 (platelet glycoprotein IIIa, antigen CD61)	UP	0.968	0.00090611
3725	JUN	jun proto-oncogene	UP	0.429	0.009899787
3885	KRT34	keratin 34, type I	UP	0.822	0.000808213

3914	LAMB3	laminin, beta 3	UP	0.597	0.00305851
3918	LAMC2	laminin, gamma 2	UP	0.429	0.001598206
3954	LETM1	leucine zipper-EF-hand containing transmembrane protein 1	UP	0.703	0.005731782
3965	LGALS9	lectin, galactoside-binding, soluble, 9	UP	0.656	0.004944348
3975	LHX1	LIM homeobox 1	UP	0.441	0.003056963
4005	LMO2	LIM domain only 2 (rhombotin-like 1)	UP	0.429	8.67632E-05
4094	MAF	v-maf avian musculoaponeurotic fibrosarcoma oncogene homolog	UP	0.478	0.000238754
4128	MAOA	monoamine oxidase A	UP	0.439	0.000497263
4188	MDFI	MyoD family inhibitor	UP	0.859	0.000903046
4318	MMP9	matrix metallopeptidase 9	UP	0.787	1.89184E-05
4490	MT1B	metallothionein 1B	UP	0.414	0.003303275
4493	MT1E	metallothionein 1E	UP	0.435	0.00579438
4501	MT1X	metallothionein 1X	UP	0.598	0.003923359
4597	MVD	mevalonate (diphospho) decarboxylase	UP	0.483	0.003253054
4599	MX1	MX dynamin-like GTPase 1	UP	1.979	9.48192E-08
4609	MYC	v-myc avian myelocytomatosis viral oncogene homolog	UP	0.442	0.002482157
4660	PPP1R12B	protein phosphatase 1, regulatory subunit 12B	UP	0.498	0.004621779
4783	NFIL3	nuclear factor, interleukin 3 regulated	UP	0.472	0.001617789
4804	NGFR	nerve growth factor receptor	UP	0.67	0.002872841
4860	PNP	purine nucleoside phosphorylase	UP	0.489	0.000411825
4861	NPAS1	neuronal PAS domain protein 1	UP	0.512	0.008804047
4938	OAS1	2'-5'-oligoadenylate synthetase 1, 40/46kDa	UP	1.404	0.000219906
4939	OAS2	2'-5'-oligoadenylate synthetase 2, 69/71kDa	UP	1.836	6.5312E-06
4940	OAS3	2'-5'-oligoadenylate synthetase 3, 100kDa	UP	0.995	0.001041852
5106	PCK2	phosphoenolpyruvate carboxykinase 2 (mitochondrial)	UP	0.507	0.005412643
5138	PDE2A	phosphodiesterase 2A, cGMP-stimulated	UP	0.466	0.009809376
5142	PDE4B	phosphodiesterase 4B, cAMP-specific	UP	0.45	0.002748583
5271	SERPINB8	serpin peptidase inhibitor, clade B (ovalbumin), member 8	UP	0.556	0.000883979
5328	PLAU	plasminogen activator, urokinase	UP	0.655	0.000469611

5329	PLAUR	plasminogen activator, urokinase receptor	UP	0.553	0.001910555
5359	PLSCR1	phospholipid scramblase 1	UP	0.509	0.008071649
5610	EIF2AK2	eukaryotic translation initiation factor 2-alpha kinase 2	UP	0.535	0.001116465
5801	PTPRR	protein tyrosine phosphatase, receptor type, R	UP	0.447	0.001189121
5814	PURB	purine-rich element binding protein B	UP	0.425	0.004773153
5820	PVT1	Pvt1 oncogene (non-protein coding)	UP	0.51	0.001028316
5997	RGS2	regulator of G-protein signaling 2	UP	0.594	0.000608022
6004	RGS16	regulator of G-protein signaling 16	UP	0.446	0.006824509
6273	S100A2	S100 calcium binding protein A2	UP	0.905	0.000383024
6346	CCL1	chemokine (C-C motif) ligand 1	UP	0.965	8.06494E-05
6348	CCL3	chemokine (C-C motif) ligand 3	UP	1.489	0.00035815
6351	CCL4	chemokine (C-C motif) ligand 4	UP	0.674	0.004371425
6352	CCL5	chemokine (C-C motif) ligand 5	UP	0.682	0.000407164
6580	SLC22A1	solute carrier family 22 (organic cation transporter), member 1	UP	0.488	0.003161816
6690	SPINK1	serine peptidase inhibitor, Kazal type 1	UP	1.745	0.000122938
6773	STAT2	signal transducer and activator of transcription 2, 113kDa	UP	0.654	0.00059094
6915	TBXA2R	thromboxane A2 receptor	UP	0.723	0.004850455
6926	TBX3	T-box 3	UP	0.699	0.000972193
6927	HNF1A	HNF1 homeobox A	UP	0.452	0.002382015
7015	TERT	telomerase reverse transcriptase	UP	0.518	0.000861879
7128	TNFAIP3	tumor necrosis factor, alpha-induced protein 3	UP	0.721	0.001552812
7130	TNFAIP6	tumor necrosis factor, alpha-induced protein 6	UP	0.418	0.002069242
7133	TNFRSF1B	tumor necrosis factor receptor superfamily, member 1B	UP	0.438	0.003915537
7185	TRAF1	TNF receptor-associated factor 1	UP	0.767	0.000261681
7186	TRAF2	TNF receptor-associated factor 2	UP	0.411	0.005752421
7262	PHLDA2	pleckstrin homology-like domain, family A, member 2	UP	0.522	0.008910579
7378	UPP1	uridine phosphorylase 1	UP	1.111	0.00024483
7421	VDR	vitamin D (1,25- dihydroxyvitamin D3) receptor	UP	0.724	0.00068437
7422	VEGFA	vascular endothelial growth factor A	UP	0.515	0.005032708

7480	WNT10B	wingless-type MMTV integration site family, member 10B	UP	0.424	0.002585313
7726	TRIM26	tripartite motif containing 26	UP	0.514	0.003584088
7784	ZP3	zona pellucida glycoprotein 3 (sperm receptor)	UP	0.648	0.002869742
7805	LAPTM5	lysosomal protein transmembrane 5	UP	0.419	0.007139311
7923	HSD17B8	hydroxysteroid (17-beta) dehydrogenase 8	UP	0.765	0.004799432
7975	MAFK	v-maf avian musculoaponeurotic fibrosarcoma oncogene homolog K	UP	0.479	0.009410999
8000	PSCA	prostate stem cell antigen	UP	0.418	0.004451974
8091	HMGA2	high mobility group AT-hook 2	UP	0.848	0.000703815
8111	GPR68	G protein-coupled receptor 68	UP	0.464	0.002720784
8241	RBM10	RNA binding motif protein 10	UP	0.688	0.004104943
8309	ACOX2	acyl-CoA oxidase 2, branched chain	UP	0.636	0.000667993
8379	MAD1L1	MAD1 mitotic arrest deficient-like 1 (yeast)	UP	0.598	0.000913109
8482	SEMA7A	semaphorin 7A, GPI membrane anchor (John Milton Hagen blood group)	UP	0.563	0.000985293
8519	IFITM1	interferon induced transmembrane protein 1	UP	0.887	8.44537E-05
8638	OASL	2'-5'-oligoadenylate synthetase-like	UP	1.444	0.000181321
8644	AKR1C3	aldo-keto reductase family 1, member C3	UP	0.448	0.003591545
8728	ADAM19	ADAM metallopeptidase domain 19	UP	0.619	0.004095829
8739	HRK	harakiri, BCL2 interacting protein	UP	0.54	0.005038744
8743	TNFSF10	tumor necrosis factor (ligand) superfamily, member 10	UP	0.742	8.39594E-05
8870	IER3	immediate early response 3	UP	0.468	0.00496659
9023	CH25H	cholesterol 25-hydroxylase	UP	0.983	0.000472963
9047	SH2D2A	SH2 domain containing 2A	UP	0.597	0.004390324
9201	DCLK1	doublecortin-like kinase 1	UP	0.552	0.007163452
9246	UBE2L6	ubiquitin-conjugating enzyme E2L 6	UP	0.431	0.000967214
9308	CD83	CD83 molecule	UP	0.571	0.001168209
9447	AIM2	absent in melanoma 2	UP	0.553	0.00288073
9450	LY86	lymphocyte antigen 86	UP	0.61	0.00205377
9454	HOMER3	homer scaffolding protein 3	UP	0.44	0.003115776
9518	GDF15	growth differentiation factor 15	UP	1.113	0.000994614

9572	NR1D1	nuclear receptor subfamily 1, group D, member 1	UP	0.447	0.005438791
9636	ISG15	ISG15 ubiquitin-like modifier	UP	1.312	2.92684E-05
9894	TELO2	telomere maintenance 2	UP	0.453	0.008597649
9935	MAFB	v-maf avian musculoaponeurotic fibrosarcoma oncogene homolog B	UP	1.242	5.18751E-05
9985	REC8	REC8 meiotic recombination protein	UP	0.47	0.008475638
10018	BCL2L11	BCL2-like 11 (apoptosis facilitator)	UP	0.481	0.000833962
10148	EBI3	Epstein-Barr virus induced 3	UP	0.412	2.34293E-05
10344	CCL26	chemokine (C-C motif) ligand 26	UP	0.59	8.51209E-05
10365	KLF2	Kruppel-like factor 2	UP	0.494	0.002609832
10379	IRF9	interferon regulatory factor 9	UP	1.55	3.91917E-05
10380	BPNT1	3'(2'), 5'-bisphosphate nucleotidase 1	UP	0.646	0.004799341
10544	PROCR	protein C receptor, endothelial	UP	0.516	0.000748583
10561	IFI44	interferon-induced protein 44	UP	1.116	0.000611629
10616	RBCK1	RanBP-type and C3HC4-type zinc finger containing 1	UP	0.413	0.005006357
10622	POLR3G	polymerase (RNA) III (DNA directed) polypeptide G (32kD)	UP	0.528	0.000387248
10673	TNFSF13B	tumor necrosis factor (ligand) superfamily, member 13b	UP	0.496	0.00535949
10675	CSPG5	chondroitin sulfate proteoglycan 5 (neuroglycan C)	UP	0.471	0.006924784
10865	ARID5A	AT rich interactive domain 5A (MRF1-like)	UP	0.504	0.001253845
10866	HCP5	HLA complex P5 (non-protein coding)	UP	0.448	0.000841456
10964	IFI44L	interferon-induced protein 44-like	UP	2.27	2.91424E-06
11095	ADAMTS8	ADAM metallopeptidase with thrombospondin type 1 motif, 8	UP	0.709	0.001025691
11132	CAPN10	calpain 10	UP	0.423	0.002144405
11274	USP18	ubiquitin specific peptidase 18	UP	0.754	0.001555652
22835	ZFP30	ZFP30 zinc finger protein	UP	0.453	0.005548823
22904	SBNO2	strawberry notch homolog 2 (Drosophila)	UP	0.501	0.005705942
22917	ZP1	zona pellucida glycoprotein 1 (sperm receptor)	UP	0.463	0.007198051
23327	NEDD4L	neural precursor cell expressed, developmentally down-regulated 4-like, E3 ubiquitin protein ligase	UP	0.496	0.008492959
23331	TTC28	tetratricopeptide repeat domain 28	UP	0.723	0.003434147

23541	SEC14L2	SEC14-like lipid binding 2	UP	0.495	0.006861084
23583	SMUG1	single-strand-selective monofunctional uracil-DNA glycosylase 1	UP	0.536	0.003833507
23586	DDX58	DEAD (Asp-Glu-Ala-Asp) box polypeptide 58	UP	0.878	0.000852455
23645	PPP1R15A	protein phosphatase 1, regulatory subunit 15A	UP	0.447	0.005447072
23677	SH3BP4	SH3-domain binding protein 4	UP	0.452	0.004697238
23764	MAFF	v-maf avian musculoaponeurotic fibrosarcoma oncogene homolog F	UP	0.516	0.002417887
23765	IL17RA	interleukin 17 receptor A	UP	0.494	0.003043009
26150	RIBC2	RIB43A domain with coiled-coils 2	UP	0.446	0.009148092
26301	GBGT1	globoside alpha-1,3-N-acetylgalactosaminyltransferase 1	UP	0.485	0.002693939
26578	OSTF1	osteoclast stimulating factor 1	UP	0.409	0.002138583
26579	MYEOV	myeloma overexpressed	UP	0.469	0.008375911
26863	RNVU1-18	RNA, variant U1 small nuclear 18	UP	0.713	0.004350229
26872	STEAP1	six transmembrane epithelial antigen of the prostate 1	UP	0.568	0.000388438
27033	ZBTB32	zinc finger and BTB domain containing 32	UP	0.789	0.001471113
27040	LAT	linker for activation of T cells	UP	0.471	0.000668381
27063	ANKRD1	ankyrin repeat domain 1 (cardiac muscle)	UP	0.48	0.009068293
27074	LAMP3	lysosomal-associated membrane protein 3	UP	0.438	0.002407763
27128	CYTH4	cytohesin 4	UP	0.431	0.008499603
29015	SLC43A3	solute carrier family 43, member 3	UP	0.447	0.001606538
29780	PARVB	parvin, beta	UP	1.015	0.00051079
29914	UBIAD1	UbiA prenyltransferase domain containing 1	UP	0.534	0.0003489
29985	SLC39A3	solute carrier family 39 (zinc transporter), member 3	UP	0.521	0.00694768
30009	TBX21	T-box 21	UP	0.521	0.00228414
30848	CTAG2	cancer/testis antigen 2	UP	0.512	0.006553982
50486	G0S2	G0/G1 switch 2	UP	0.517	0.002748352
50964	SOST	sclerostin	UP	0.714	0.001613171
51129	ANGPTL4	angiopoietin-like 4	UP	1.217	0.00193021
51148	CERCAM	cerebral endothelial cell adhesion molecule	UP	0.553	0.000725271
51176	LEF1	lymphoid enhancer-binding factor 1	UP	0.672	0.000448244

51296	SLC15A3	solute carrier family 15 (oligopeptide transporter), member 3	UP	0.718	0.000685985
51299	NRN1	neuritin 1	UP	0.462	0.006071523
51513	ETV7	ets variant 7	UP	0.467	0.009343652
51561	IL23A	interleukin 23, alpha subunit p19	UP	1.247	0.000134631
51655	RASD1	RAS, dexamethasone-induced 1	UP	0.737	0.000250692
51676	ASB2	ankyrin repeat and SOCS box containing 2	UP	0.461	0.008863725
51710	ZNF44	zinc finger protein 44	UP	0.482	0.005514977
54210	TREM1	triggering receptor expressed on myeloid cells 1	UP	0.598	0.007604361
54438	GFOD1	glucose-fructose oxidoreductase domain containing 1	UP	0.529	0.006765315
54739	XAF1	XIAP associated factor 1	UP	1.166	0.000133788
54742	LY6K	lymphocyte antigen 6 complex, locus K	UP	0.477	0.002217572
54894	RNF43	ring finger protein 43	UP	0.504	0.006413268
54993	ZSCAN2	zinc finger and SCAN domain containing 2	UP	0.775	0.004367427
55007	FAM118A	family with sequence similarity 118, member A	UP	0.766	0.001752776
55076	TMEM45A	transmembrane protein 45A	UP	0.613	0.001676429
55359	STYK1	serine/threonine/tyrosine kinase 1	UP	0.58	0.002938799
55733	HHAT	hedgehog acyltransferase	UP	0.578	0.002687207
56829	ZC3HAV1	zinc finger CCCH-type, antiviral 1	UP	0.458	0.000779642
56905	C15orf39	chromosome 15 open reading frame 39	UP	0.465	0.003554214
56952	PRTFDC1	phosphoribosyl transferase domain containing 1	UP	0.433	0.003280721
57026	PDXP	pyridoxal (pyridoxine, vitamin B6) phosphatase	UP	0.429	0.00308955
57214	CEMIP	cell migration inducing protein, hyaluronan binding	UP	0.559	0.008417914
57608	KIAA1462	KIAA1462	UP	0.563	0.000483556
57642	COL20A1	collagen, type XX, alpha 1	UP	0.681	0.002568975
57794	SUGP1	SURP and G patch domain containing 1	UP	0.43	0.002168874
60343	FAM3A	family with sequence similarity 3, member A	UP	0.483	0.006138923
60481	ELOVL5	ELOVL fatty acid elongase 5	UP	0.465	0.007230939
60673	ATG101	autophagy related 101	UP	0.449	0.001085127
64108	RTP4	receptor (chemosensory) transporter protein 4	UP	0.662	0.003468206

64109	CRLF2	cytokine receptor-like factor 2	UP	0.78	0.003035333
64409	WBSCR17	Williams-Beuren syndrome chromosome region 17	UP	0.421	0.006417532
64600	PLA2G2F	phospholipase A2, group IIF	UP	0.611	0.003593625
64761	PARP12	poly (ADP-ribose) polymerase family, member 12	UP	0.452	0.002831303
64794	DDX31	DEAD (Asp-Glu-Ala-Asp) box polypeptide 31	UP	0.474	0.004646483
78986	DUSP26	dual specificity phosphatase 26 (putative)	UP	0.624	0.000325345
79015	LINC01260	long intergenic non-protein coding RNA 1260	UP	0.522	0.006562587
79132	DHX58	DEXH (Asp-Glu-X-His) box polypeptide 58	UP	0.433	0.003251763
79155	TNIP2	TNFAIP3 interacting protein 2	UP	0.493	0.005952339
79156	PLEKHF1	pleckstrin homology domain containing, family F (with FYVE domain) member 1	UP	0.576	0.005075996
79160	LOC79160	uncharacterized LOC79160	UP	0.497	0.00699616
79413	ZBED2	zinc finger, BED-type containing 2	UP	0.743	8.60392E-05
79709	COLGALT1	collagen beta(1-O)galactosyltransferase 1	UP	0.411	0.002423137
79754	ASB13	ankyrin repeat and SOCS box containing 13	UP	0.427	0.009866016
79827	CLMP	CXADR-like membrane protein	UP	0.498	0.001882997
79847	MFSD13A	major facilitator superfamily domain containing 13A	UP	0.528	0.003342753
79933	SYNPO2L	synaptopodin 2-like	UP	0.789	0.001860447
79966	SCD5	stearoyl-CoA desaturase 5	UP	0.486	0.000488525
81621	KAZALD1	Kazal-type serine peptidase inhibitor domain 1	UP	0.461	0.005444243
83538	TTC25	tetratricopeptide repeat domain 25	UP	0.528	0.001687393
83666	PARP9	poly (ADP-ribose) polymerase family, member 9	UP	0.518	0.00589137
83895	KRTAP1-5	keratin associated protein 1-5	UP	0.942	0.0016068
84065	TMEM222	transmembrane protein 222	UP	0.428	0.003433116
84419	C15orf48	chromosome 15 open reading frame 48	UP	0.575	0.003121787
84445	LZTS2	leucine zipper, putative tumor suppressor 2	UP	0.617	0.005154769
84717	HDGFRP2	hepatoma-derived growth factor-related protein 2	UP	0.731	0.00209223
84791	LINC00467	long intergenic non-protein coding RNA 467	UP	0.425	0.007730886
84879	MFSD2A	major facilitator superfamily domain containing 2A	UP	0.53	0.00370377
84951	TNS4	tensin 4	UP	0.63	0.006243667

85463	ZC3H12C	zinc finger CCCH-type containing 12C	UP	0.493	0.003718548
91543	RSAD2	radical S-adenosyl methionine domain containing 2	UP	1.861	5.54746E-06
92579	G6PC3	glucose 6 phosphatase, catalytic, 3	UP	0.409	0.00040376
92659	MAFG-AS1	MAFG antisense RNA 1 (head to head)	UP	0.474	0.006367062
93082	NEURL3	neuralized E3 ubiquitin protein ligase 3	UP	0.455	0.009000127
93343	MVB12A	multivesicular body subunit 12A	UP	0.406	0.001144348
93429	DKFZp434J0226	uncharacterized LOC93429	UP	0.54	0.000978053
94120	SYTL3	synaptotagmin-like 3	UP	0.736	0.003206778
94240	EPSTI1	epithelial stromal interaction 1 (breast)	UP	0.628	0.000456154
112597	LINC00152	long intergenic non-protein coding RNA 152	UP	0.445	0.005769483
114780	PKD1L2	polycystic kidney disease 1-like 2 (gene/pseudogene)	UP	1.061	0.001500352
114801	TMEM200A	transmembrane protein 200A	UP	0.548	0.001380445
114907	FBXO32	F-box protein 32	UP	0.675	0.0032363
115123	42066	membrane-associated ring finger (C3HC4) 3, E3 ubiquitin protein ligase	UP	0.541	0.001303634
115265	DDIT4L	DNA-damage-inducible transcript 4-like	UP	0.621	0.006666432
116071	BATF2	basic leucine zipper transcription factor, ATF-like 2	UP	0.544	0.002613168
121268	RHEBL1	Ras homolog enriched in brain like 1	UP	0.538	0.007321553
122509	IFI27L1	interferon, alpha-inducible protein 27-like 1	UP	0.543	9.41567E-05
125488	TTC39C	tetratricopeptide repeat domain 39C	UP	0.459	0.007240138
125965	COX6B2	cytochrome c oxidase subunit VIb polypeptide 2 (testis)	UP	0.705	0.003829701
128434	VSTM2L	V-set and transmembrane domain containing 2 like	UP	0.453	0.00556139
129607	CMPK2	cytidine monophosphate (UMP-CMP) kinase 2, mitochondrial	UP	0.752	0.000239827
130827	TMEM182	transmembrane protein 182	UP	0.474	0.002685755
140686	WFDC3	WAP four-disulfide core domain 3	UP	0.879	0.000236502
145200	LINC00239	long intergenic non-protein coding RNA 239	UP	0.605	0.00117586
145694	LOC145694	uncharacterized LOC145694	UP	0.477	0.008739228
146512	FLJ30679	uncharacterized protein FLJ30679	UP	0.914	0.001858306
147920	IGFL2	IGF-like family member 2	UP	0.502	0.00084027
149773	APCDD1L-AS1	APCDD1L antisense RNA 1 (head to head)	UP	0.773	0.000912105

149840	C20orf196	chromosome 20 open reading frame 196	UP	0.958	0.001450939
152302	CIDECP	cell death-inducing DFFA-like effector c pseudogene	UP	0.43	0.003220079
152816	C4orf26	chromosome 4 open reading frame 26	UP	0.816	0.002468327
157627	LINC00599	long intergenic non-protein coding RNA 599	UP	1.021	0.001537471
158511	CSAG1	chondrosarcoma associated gene 1	UP	0.856	0.000429789
160428	ALDH1L2	aldehyde dehydrogenase 1 family, member L2	UP	0.656	0.001198151
169841	ZNF169	zinc finger protein 169	UP	0.568	0.001726307
201651	AADACP1	arylacetamide deacetylase pseudogene 1	UP	0.579	0.003134782
203413	CT83	cancer/testis antigen 83	UP	0.62	0.001411346
219855	SLC37A2	solute carrier family 37 (glucose-6-phosphate transporter), member 2	UP	0.535	0.001322868
219874	OR6T1	olfactory receptor, family 6, subfamily T, member 1	UP	0.461	0.001385133
221476	PI16	peptidase inhibitor 16	UP	0.484	0.000384789
222255	ATXN7L1	ataxin 7-like 1	UP	0.429	0.009675221
246721	POLR2J2	polymerase (RNA) II (DNA directed) polypeptide J2	UP	0.832	0.003275104
253832	ZDHHC20	zinc finger, DHHC-type containing 20	UP	0.619	0.003341309
256227	STEAP1B	STEAP family member 1B	UP	0.682	0.004705271
256979	SUN3	Sad1 and UNC84 domain containing 3	UP	0.619	0.000492346
282616	IFNL2	interferon, lambda 2	UP	0.718	0.002503324
283131	NEAT1	nuclear paraspeckle assembly transcript 1 (non-protein coding)	UP	0.731	0.00126943
283212	KLHL35	kelch-like family member 35	UP	0.531	0.000665371
283248	RCOR2	REST corepressor 2	UP	0.422	0.009229888
284276	LINC00908	long intergenic non-protein coding RNA 908	UP	0.484	0.001860231
284613	CYB561D1	cytochrome b561 family, member D1	UP	0.433	0.003077424
339168	TMEM95	transmembrane protein 95	UP	0.462	0.008985674
340390	WDR97	WD repeat domain 97	UP	0.83	0.003213046
348761	SPATA3-AS1	SPATA3 antisense RNA 1 (head to head)	UP	1.271	0.001048289
348938	NIPAL4	NIPA-like domain containing 4	UP	0.546	0.007835691
349075	ZNF713	zinc finger protein 713	UP	0.686	0.005480916
373856	USP41	ubiquitin specific peptidase 41	UP	0.495	0.006642796

374897	SBSN	suprabasin	UP	0.716	0.000417181
375295	LINC01116	long intergenic non-protein coding RNA 1116	UP	0.57	0.006320873
387763	C11orf96	chromosome 11 open reading frame 96	UP	0.948	0.001850121
388394	RPRML	reprimo-like	UP	0.556	0.002174105
389332	LOC389332	uncharacterized LOC389332	UP	1.002	0.001032926
390748	PABPN1L	poly(A) binding protein, nuclear 1-like (cytoplasmic)	UP	0.779	0.001703085
400745	SH2D5	SH2 domain containing 5	UP	0.494	0.001890217
401317	LOC401317	uncharacterized LOC401317	UP	0.607	0.003586408
401357	LOC401357	uncharacterized LOC401357	UP	0.563	0.002840644
401647	GOLGA7B	golgin A7 family, member B	UP	0.678	0.000810652
541471	MIR4435-2HG	MIR4435-2 host gene	UP	0.507	0.00626398
642691	FLJ37786	uncharacterized LOC642691	UP	0.718	0.000746325
645687	LINC00520	long intergenic non-protein coding RNA 520	UP	0.486	0.006756929
654346	LGALS9C	lectin, galactoside-binding, soluble, 9C	UP	0.67	0.001603734
728192	LINC00460	long intergenic non-protein coding RNA 460	UP	0.486	0.001162996
728461	#N/A	#N/A	UP	0.715	0.003558759
729013	ZBED5-AS1	ZBED5 antisense RNA 1	UP	0.415	0.004995431
730755	KRTAP2-3	keratin associated protein 2-3	UP	0.875	0.003921539
100126781	SNAR-F	small ILF3/NF90-associated RNA F	UP	0.524	0.009249697
100130171	LOC100130171	hematological and neurological expressed 1 pseudogene	UP	0.42	0.007928769
100130935	CSAG4	CSAG family, member 4 (pseudogene)	UP	0.846	0.000456267
100130938	#N/A	#N/A	UP	0.916	0.000523363
100131390	SP9	Sp9 transcription factor	UP	0.446	0.00081877
100132116	ACTA2-AS1	ACTA2 antisense RNA 1	UP	0.414	0.008617078
100132368	LOC100132368	uncharacterized LOC100132368	UP	0.432	0.00832414
100133171	#N/A	#N/A	UP	0.501	0.005696674
100216001	LINC00704	long intergenic non-protein coding RNA 704	UP	0.497	0.008577842
100271722	LINC00899	long intergenic non-protein coding RNA 899	UP	0.494	0.009282842
100287177	EML2-AS1	EML2 antisense RNA 1	UP	0.447	0.000270744

100506178	LOC100506178	uncharacterized LOC100506178	UP	0.75	0.001350528
100506190	LINC00963	long intergenic non-protein coding RNA 963	UP	0.732	0.003261333
100506377	LINC00973	long intergenic non-protein coding RNA 973	UP	0.905	0.000429916
100506403	LOC100506403	uncharacterized LOC100506403	UP	0.567	0.000654366
100506718	LOC100506718	uncharacterized LOC100506718	UP	0.604	0.000268821
100506955	LOC100506955	cancer/testis antigen 83 pseudogene	UP	0.411	0.005652485
100507144	LOC100507144	uncharacterized LOC100507144	UP	0.543	0.003330247
100507410	C1QTNF1-AS1	C1QTNF1 antisense RNA 1	UP	0.683	0.000338433
100509445	#N/A	#N/A	UP	0.472	0.001380868
100652988	LINC00702	long intergenic non-protein coding RNA 702	UP	0.656	0.001809759
100996267	#N/A	#N/A	UP	0.869	0.003944323
101059954	LOC101059954	uncharacterized LOC101059954	UP	0.858	0.000155571
101927413	LOC101927413	uncharacterized LOC101927413	UP	0.445	0.007977155
101927440	LOC101927440	uncharacterized LOC101927440	UP	0.41	0.009263877
101927528	LINC01204	long intergenic non-protein coding RNA 1204	UP	0.634	0.00561219
101928841	LOC101928841	collagen alpha-1(II) chain-like	UP	0.596	0.002042951
101930294	LOC101930294	uncharacterized LOC101930294	UP	1.346	0.000453769
90	ACVR1	activin A receptor, type I	DOWN	-0.522	0.00954461
250	ALPP	alkaline phosphatase, placental	DOWN	-0.99	6.2378E-05
594	BCKDHB	branched chain keto acid dehydrogenase E1, beta polypeptide	DOWN	-0.613	0.002515332
627	BDNF	brain-derived neurotrophic factor	DOWN	-0.635	0.002671154
648	BMI1	BMI1 proto-oncogene, polycomb ring finger	DOWN	-0.453	0.008429462
652	BMP4	bone morphogenetic protein 4	DOWN	-0.644	0.000770373
688	KLF5	Kruppel-like factor 5 (intestinal)	DOWN	-0.448	0.009236583
821	CANX	calnexin	DOWN	-0.522	0.009154841
827	CAPN6	calpain 6	DOWN	-0.468	0.001651905
829	CAPZA1	capping protein (actin filament) muscle Z-line, alpha 1	DOWN	-0.559	0.005024038
857	CAV1	caveolin 1, caveolae protein, 22kDa	DOWN	-0.553	0.005815594
891	CCNB1	cyclin B1	DOWN	-0.485	0.003889309

900	CCNG1	cyclin G1	DOWN	-0.509	0.009799775
999	CDH1	cadherin 1, type 1	DOWN	-0.554	0.002316668
1105	CHD1	chromodomain helicase DNA binding protein 1	DOWN	-0.436	0.003681049
1176	AP3S1	adaptor-related protein complex 3, sigma 1 subunit	DOWN	-0.608	0.002026352
1295	COL8A1	collagen, type VIII, alpha 1	DOWN	-0.685	0.000534844
1495	CTNNA1	catenin (cadherin-associated protein), alpha 1, 102kDa	DOWN	-0.599	0.006379696
1602	DACH1	dachshund family transcription factor 1	DOWN	-0.44	0.002899272
1739	DLG1	discs, large homolog 1 (Drosophila)	DOWN	-0.566	0.000152069
1748	DLX4	distal-less homeobox 4	DOWN	-0.512	0.001026276
1832	DSP	desmoplakin	DOWN	-0.55	0.006811403
1854	DUT	deoxyuridine triphosphatase	DOWN	-0.455	0.007721352
1875	E2F5	E2F transcription factor 5, p130-binding	DOWN	-0.447	0.004730383
1894	ECT2	epithelial cell transforming 2	DOWN	-0.678	0.004580381
1917	EEF1A2	eukaryotic translation elongation factor 1 alpha 2	DOWN	-0.553	0.006863051
1982	EIF4G2	eukaryotic translation initiation factor 4 gamma, 2	DOWN	-0.45	0.006910985
2107	ETF1	eukaryotic translation termination factor 1	DOWN	-0.439	0.008476337
2114	ETS2	v-ets avian erythroblastosis virus E26 oncogene homolog 2	DOWN	-0.452	0.007336888
2185	PTK2B	protein tyrosine kinase 2 beta	DOWN	-0.437	0.004658602
2353	FOS	FBJ murine osteosarcoma viral oncogene homolog	DOWN	-0.436	0.009210672
2562	GABRB3	gamma-aminobutyric acid (GABA) A receptor, beta 3	DOWN	-0.48	0.006263141
2744	GLS	glutaminase	DOWN	-0.507	0.008164989
2771	GNAI2	guanine nucleotide binding protein (G protein),<br>alpha inhibiting activity polypeptide 2	DOWN	-0.457	0.001323269
2778	GNAS	GNAS complex locus	DOWN	-0.483	0.0087854
2969	GTF2I	general transcription factor IIi	DOWN	-0.536	0.002983154
2982	GUCY1A3	guanylate cyclase 1, soluble, alpha 3	DOWN	-0.446	0.003690547
3024	HIST1H1A	histone cluster 1, H1a	DOWN	-0.594	0.002112432
3091	HIF1A	hypoxia inducible factor 1, alpha subunit<br>(basic helix-loop-helix transcription factor)	DOWN	-0.559	0.00253281

3149	HMGB3	high mobility group box 3	DOWN	-0.464	0.005703159
3658	IREB2	iron-responsive element binding protein 2	DOWN	-0.441	0.007579852
3667	IRS1	insulin receptor substrate 1	DOWN	-0.505	0.007530719
3842	TNPO1	transportin 1	DOWN	-0.45	0.003119265
3895	KTN1	kinectin 1 (kinesin receptor)	DOWN	-0.435	0.007325956
3912	LAMB1	laminin, beta 1	DOWN	-0.575	0.006871632
3977	LIFR	leukemia inhibitory factor receptor alpha	DOWN	-0.723	0.004400521
3993	LLGL2	lethal giant larvae homolog 2 (Drosophila)	DOWN	-0.484	0.003925776
4046	LSP1	lymphocyte-specific protein 1	DOWN	-0.736	0.000723552
4087	SMAD2	SMAD family member 2	DOWN	-0.439	0.008535929
4131	MAP1B	microtubule-associated protein 1B	DOWN	-0.434	0.004916016
4204	MECP2	methyl CpG binding protein 2	DOWN	-0.461	0.003160324
4217	MAP3K5	mitogen-activated protein kinase kinase kinase 5	DOWN	-0.422	0.001371225
4289	MKLN1	muskelin 1, intracellular mediator containing kelch motifs	DOWN	-0.469	0.005436244
4508	ATP6	ATP synthase F0 subunit 6	DOWN	-0.526	0.004580115
4534	MTM1	myotubularin 1	DOWN	-0.467	0.008896854
4539	ND4L	NADH dehydrogenase, subunit 4L (complex I)	DOWN	-0.485	0.002430498
4683	NBN	nibrin	DOWN	-0.467	0.00205352
5042	PABPC3	poly(A) binding protein, cytoplasmic 3	DOWN	-0.406	0.00466387
5125	PCSK5	proprotein convertase subtilisin/kexin type 5	DOWN	-0.517	6.56651E-06
5174	PDZK1	PDZ domain containing 1	DOWN	-0.701	0.002335125
5189	PEX1	peroxisomal biogenesis factor 1	DOWN	-0.416	0.009991525
5205	ATP8B1	ATPase, aminophospholipid transporter, class I, type 8B, member 1	DOWN	-0.518	0.004750039
5290	PIK3CA	phosphatidylinositol-4,5-bisphosphate 3-kinase, catalytic subunit alpha	DOWN	-0.408	0.000582822
5493	PPL	periplakin	DOWN	-0.531	0.002352156
5597	MAPK6	mitogen-activated protein kinase 6	DOWN	-0.42	0.001681828
5650	KLK7	kallikrein-related peptidase 7	DOWN	-0.779	0.004529034
5918	RARRES1	retinoic acid receptor responder (tazarotene induced) 1	DOWN	-0.773	0.001219814
5934	RBL2	retinoblastoma-like 2	DOWN	-0.407	0.005750111

6157	RPL27A	ribosomal protein L27a	DOWN	-0.478	0.004681443
6194	RPS6	ribosomal protein S6	DOWN	-0.413	0.009459515
6272	SORT1	sortilin 1	DOWN	-0.586	0.005225548
6311	ATXN2	ataxin 2	DOWN	-0.414	0.000566643
6326	SCN2A	sodium channel, voltage gated, type II alpha subunit	DOWN	-0.429	0.005578795
6398	SECTM1	secreted and transmembrane 1	DOWN	-0.456	0.001890575
6414	SEPP1	selenoprotein P, plasma, 1	DOWN	-0.483	0.002382989
6429	SRSF4	serine/arginine-rich splicing factor 4	DOWN	-0.506	0.002679535
6584	SLC22A5	solute carrier family 22 (organic cation/carnitine transporter), member 5	DOWN	-0.444	0.001533137
6596	HLTF	helicase-like transcription factor	DOWN	-0.452	0.003105298
6751	SSTR1	somatostatin receptor 1	DOWN	-0.457	0.000329079
6801	STRN	striatin, calmodulin binding protein	DOWN	-0.487	0.001848294
6872	TAF1	TAF1 RNA polymerase II,<br>TATA box binding protein (TBP)-associated factor, 250kDa	DOWN	-0.409	0.00119799
6874	TAF4	TAF4 RNA polymerase II,<br>TATA box binding protein (TBP)-associated factor, 135kDa	DOWN	-0.539	0.001996124
6996	TDG	thymine DNA glycosylase	DOWN	-0.491	0.005794895
7010	TEK	TEK tyrosine kinase, endothelial	DOWN	-0.696	0.000718404
7020	TFAP2A	transcription factor AP-2 alpha (activating enhancer binding protein 2 alpha)	DOWN	-0.464	0.00151374
7112	TMPO	thymopoietin	DOWN	-0.507	0.006881813
7123	CLEC3B	C-type lectin domain family 3, member B	DOWN	-0.548	0.000662767
7140	TNNT3	troponin T type 3 (skeletal, fast)	DOWN	-0.594	0.000807374
7153	TOP2A	topoisomerase (DNA) II alpha	DOWN	-0.592	0.002772869
7204	TRIO	trio Rho guanine nucleotide exchange factor	DOWN	-0.48	0.005727578
7306	TYRP1	tyrosinase-related protein 1	DOWN	-0.54	0.000386275
7750	ZMYM2	zinc finger, MYM-type 2	DOWN	-0.516	0.006707127
8554	PIAS1	protein inhibitor of activated STAT, 1	DOWN	-0.434	0.000426356
8581	LY6D	lymphocyte antigen 6 complex, locus D	DOWN	-0.495	0.004279818
8654	PDE5A	phosphodiesterase 5A, cGMP-specific	DOWN	-0.888	0.002661207

8786	RGS11	regulator of G-protein signaling 11	DOWN	-0.519	0.003111745
9037	SEMA5A	sema domain, seven thrombospondin repeats (type 1 and type 1-like), transmembrane domain (TM) and short cytoplasmic domain, (semaphorin) 5A	DOWN	-0.844	0.00079043
9126	SMC3	structural maintenance of chromosomes 3	DOWN	-0.518	0.006527597
9397	NMT2	N-myristoyltransferase 2	DOWN	-0.41	0.007116323
9439	MED23	mediator complex subunit 23	DOWN	-0.582	0.005917621
9517	SPTLC2	serine palmitoyltransferase, long chain base subunit 2	DOWN	-0.496	0.006433528
9562	MINPP1	multiple inositol-polyphosphate phosphatase 1	DOWN	-0.458	0.003406187
9615	GDA	guanine deaminase	DOWN	-0.488	0.005490091
9627	SNCAIP	synuclein, alpha interacting protein	DOWN	-0.525	0.007572186
9678	PHF14	PHD finger protein 14	DOWN	-0.474	0.008460005
9750	FAM65B	family with sequence similarity 65, member B	DOWN	-0.57	0.005337615
9793	CKAP5	cytoskeleton associated protein 5	DOWN	-0.44	0.003026093
9847	C2CD5	C2 calcium-dependent domain containing 5	DOWN	-0.413	0.002322721
9859	CEP170	centrosomal protein 170kDa	DOWN	-0.444	0.007883099
9883	POM121	POM121 transmembrane nucleoporin	DOWN	-0.438	0.008949842
9897	KIAA0196	KIAA0196	DOWN	-0.409	0.005868784
9905	SGSM2	small G protein signaling modulator 2	DOWN	-0.493	0.002498011
9915	ARNT2	aryl-hydrocarbon receptor nuclear translocator 2	DOWN	-0.412	0.008191073
9918	NCAPD2	non-SMC condensin I complex, subunit D2	DOWN	-0.464	0.004438434
9982	FGFBP1	fibroblast growth factor binding protein 1	DOWN	-0.438	0.002410605
10042	HMGXB4	HMG box domain containing 4	DOWN	-0.463	0.005133091
10051	SMC4	structural maintenance of chromosomes 4	DOWN	-0.632	0.00193368
10129	FRY	furry homolog (Drosophila)	DOWN	-0.472	0.000941093
10198	MPHOSPH9	M-phase phosphoprotein 9	DOWN	-0.416	0.00297132
10207	INADL	InaD-like (Drosophila)	DOWN	-0.543	0.000369015
10314	LANCL1	LanC lantibiotic synthetase component C-like 1 (bacterial)	DOWN	-0.508	0.0055559
10404	CPQ	carboxypeptidase Q	DOWN	-0.476	0.004424614
10446	LRRN2	leucine rich repeat neuronal 2	DOWN	-0.464	0.001760638

10486	CAP2	CAP, adenylate cyclase-associated protein, 2 (yeast)	DOWN	-0.534	0.003726179
10492	SYNCRIP	synaptotagmin binding, cytoplasmic RNA interacting protein	DOWN	-0.458	0.00579037
10529	NEBL	nebulette	DOWN	-0.518	0.002719099
10614	HEXIM1	hexamethylene bis-acetamide inducible 1	DOWN	-0.514	0.002800129
10725	NFAT5	nuclear factor of activated T-cells 5, tonicity-responsive	DOWN	-0.678	0.004927325
10733	PLK4	polo-like kinase 4	DOWN	-0.575	0.007396889
10772	SRSF10	serine/arginine-rich splicing factor 10	DOWN	-0.41	0.004881557
10787	NCKAP1	NCK-associated protein 1	DOWN	-0.509	0.0040743
10892	MALT1	MALT1 paracaspase	DOWN	-0.457	0.008925147
10935	PRDX3	peroxiredoxin 3	DOWN	-0.442	5.10068E-05
10983	CCNI	cyclin I	DOWN	-0.439	0.009016401
11001	SLC27A2	solute carrier family 27 (fatty acid transporter), member 2	DOWN	-0.417	0.008346073
11037	STON1	stonin 1	DOWN	-0.696	0.001185828
11076	TPPP	tubulin polymerization promoting protein	DOWN	-0.73	0.000458438
11309	SLCO2B1	solute carrier organic anion transporter family, member 2B1	DOWN	-0.517	0.001542047
22829	NLGN4Y	neuroligin 4, Y-linked	DOWN	-0.484	0.003419094
22906	TRAK1	trafficking protein, kinesin binding 1	DOWN	-0.443	0.008993118
23015	GOLGA8A	golgin A8 family, member A	DOWN	-0.575	0.000550861
23126	POGZ	pogo transposable element with ZNF domain	DOWN	-0.424	0.007962174
23151	GRAMD4	GRAM domain containing 4	DOWN	-0.425	0.000471017
23161	SNX13	sorting nexin 13	DOWN	-0.523	0.002648512
23174	ZCCHC14	zinc finger, CCHC domain containing 14	DOWN	-0.438	0.00827582
23199	GSE1	Gse1 coiled-coil protein	DOWN	-0.438	0.003949805
23279	NUP160	nucleoporin 160kDa	DOWN	-0.434	0.008601863
23366	KIAA0895	KIAA0895	DOWN	-0.416	0.005301824
23468	CBX5	chromobox homolog 5	DOWN	-0.408	0.009123039
23527	ACAP2	ArfGAP with coiled-coil, ankyrin repeat and PH domains 2	DOWN	-0.532	0.007622615
23596	OPN3	opsin 3	DOWN	-0.752	0.001218491
25836	NIPBL	Nipped-B homolog (Drosophila)	DOWN	-0.554	0.005473599

26002	MOXD1	monooxygenase, DBH-like 1	DOWN	-0.436	0.001268612
26049	FAM169A	family with sequence similarity 169, member A	DOWN	-0.551	0.006398926
26060	APPL1	adaptor protein, phosphotyrosine interaction, PH domain and leucine zipper containing 1	DOWN	-0.453	0.005843574
26149	ZNF658	zinc finger protein 658	DOWN	-0.532	0.000101567
26286	ARFGAP3	ADP-ribosylation factor GTPase activating protein 3	DOWN	-0.429	0.004469403
27295	PDLIM3	PDZ and LIM domain 3	DOWN	-0.473	0.004515497
27327	TNRC6A	trinucleotide repeat containing 6A	DOWN	-0.554	0.007165186
27347	STK39	serine threonine kinase 39	DOWN	-0.435	0.000921371
50804	MYEF2	myelin expression factor 2	DOWN	-0.576	0.001572203
51326	ARL17A	ADP-ribosylation factor-like 17A	DOWN	-0.591	0.001015847
51455	REV1	REV1, polymerase (DNA directed)	DOWN	-0.443	0.004746457
51742	ARID4B	AT rich interactive domain 4B (RBP1-like)	DOWN	-0.407	0.001013619
53405	CLIC5	chloride intracellular channel 5	DOWN	-0.434	0.001032494
54014	BRWD1	bromodomain and WD repeat domain containing 1	DOWN	-0.407	0.006375575
54101	RIPK4	receptor-interacting serine-threonine kinase 4	DOWN	-0.472	0.001319287
54502	RBM47	RNA binding motif protein 47	DOWN	-0.6	0.001032666
54674	LRRN3	leucine rich repeat neuronal 3	DOWN	-1.156	0.000284109
54765	TRIM44	tripartite motif containing 44	DOWN	-0.415	0.004716162
54799	MBTD1	mbt domain containing 1	DOWN	-0.504	0.002255434
54821	ERCC6L	excision repair cross-complementation group 6-like	DOWN	-0.426	0.009361757
54873	PALMD	palmdelphin	DOWN	-0.873	0.000776497
55002	TMCO3	transmembrane and coiled-coil domains 3	DOWN	-0.521	0.008427486
55137	FIGN	fidgetin	DOWN	-0.424	0.004414398
55198	APPL2	adaptor protein, phosphotyrosine interaction, PH domain and leucine zipper containing 2	DOWN	-0.426	0.009437086
55322	C5orf22	chromosome 5 open reading frame 22	DOWN	-0.497	0.004521257
55342	STRBP	spermatid perinuclear RNA binding protein	DOWN	-0.49	0.001282892
55607	PPP1R9A	protein phosphatase 1, regulatory subunit 9A	DOWN	-0.559	0.001751129

55623	THUMPD1	THUMP domain containing 1	DOWN	-0.504	3.61186E-05
55635	DEPDC1	DEP domain containing 1	DOWN	-0.411	0.008695757
55806	HR	hair growth associated	DOWN	-0.499	0.002674747
55814	BDP1	B double prime 1, subunit of RNA polymerase III transcription initiation factor IIII	DOWN	-0.476	0.005139135
56267	CCBL2	cysteine conjugate-beta lyase 2	DOWN	-0.417	0.000379976
56916	SMARCAD1	SWI/SNF-related, matrix-associated actin-dependent regulator of chromatin, subfamily a, containing DEAD/H box 1	DOWN	-0.474	0.005638247
57016	AKR1B10	aldo-keto reductase family 1, member B10 (aldose reductase)	DOWN	-0.413	0.008216413
57181	SLC39A10	solute carrier family 39 (zinc transporter), member 10	DOWN	-0.569	0.006949669
57182	ANKRD50	ankyrin repeat domain 50	DOWN	-0.624	0.001003983
57460	PPM1H	protein phosphatase, Mg2+/Mn2+ dependent, 1H	DOWN	-0.425	0.0058681
57489	ODF2L	outer dense fiber of sperm tails 2-like	DOWN	-0.515	0.003465327
57491	AHRR	aryl-hydrocarbon receptor repressor	DOWN	-0.665	0.000744913
57493	HEG1	heart development protein with EGF-like domains 1	DOWN	-0.499	0.001807393
57532	NUFIP2	nuclear fragile X mental retardation protein interacting protein 2	DOWN	-0.622	0.004925422
57554	LRRC7	leucine rich repeat containing 7	DOWN	-0.62	0.002584397
57639	CCDC146	coiled-coil domain containing 146	DOWN	-0.449	0.001292187
57649	PHF12	PHD finger protein 12	DOWN	-0.547	0.008762755
57703	CWC22	CWC22 spliceosome-associated protein	DOWN	-0.495	0.006127507
63027	SLC22A23	solute carrier family 22, member 23	DOWN	-0.523	0.002817142
63939	FAM217B	family with sequence similarity 217, member B	DOWN	-0.551	0.001720265
64393	ZMAT3	zinc finger, matrin-type 3	DOWN	-0.488	0.002191901
64710	NUCKS1	nuclear casein kinase and cyclin-dependent kinase substrate 1	DOWN	-0.445	0.009939968
64746	ACBD3	acyl-CoA binding domain containing 3	DOWN	-0.43	0.007020145
64759	TNS3	tensin 3	DOWN	-0.6	0.003909754
64781	CERK	ceramide kinase	DOWN	-0.475	0.006685531
64921	CASD1	CAS1 domain containing 1	DOWN	-0.468	0.001271912
79109	MAPKAP1	mitogen-activated protein kinase associated protein 1	DOWN	-0.444	0.005867772
79684	MSANTD2	Myb/SANT-like DNA-binding domain containing 2	DOWN	-0.552	0.005076189

79801	SHCBP1	SHC SH2-domain binding protein 1	DOWN	-0.417	0.004421332
79844	ZDHHC11	zinc finger, DHHC-type containing 11	DOWN	-0.551	0.003574468
79937	CNTNAP3	contactin associated protein-like 3	DOWN	-0.695	0.000520474
80063	ATF7IP2	activating transcription factor 7 interacting protein 2	DOWN	-0.594	0.002313597
80312	TET1	tet methylcytosine dioxygenase 1	DOWN	-0.513	0.00044902
80817	CEP44	centrosomal protein 44kDa	DOWN	-0.459	0.00038249
81493	SYNC	syncoilin, intermediate filament protein	DOWN	-0.635	0.002813521
81610	FAM83D	family with sequence similarity 83, member D	DOWN	-0.468	0.008604164
81794	ADAMTS10	ADAM metallopeptidase with thrombospondin type 1 motif, 10	DOWN	-0.417	0.009767256
84162	KIAA1109	KIAA1109	DOWN	-0.539	0.000385126
84612	PARD6B	par-6 family cell polarity regulator beta	DOWN	-0.491	0.00869047
84962	AJUBA	ajuba LIM protein	DOWN	-0.478	0.009575878
89846	FGD3	FYVE, RhoGEF and PH domain containing 3	DOWN	-0.756	0.000857208
91948	LINC00923	long intergenic non-protein coding RNA 923	DOWN	-0.411	0.00801708
92070	CTBP1-AS2	CTBP1 antisense RNA 2 (head to head)	DOWN	-0.418	0.009144567
92154	MTSS1L	metastasis suppressor 1-like	DOWN	-0.478	0.003988949
112476	PRRT2	proline-rich transmembrane protein 2	DOWN	-0.409	0.000913186
114800	CCDC85A	coiled-coil domain containing 85A	DOWN	-0.506	0.000938512
116064	LRRC58	leucine rich repeat containing 58	DOWN	-0.685	0.003629638
123606	NIPA1	non imprinted in Prader-Willi/Angelman syndrome 1	DOWN	-0.423	0.008928916
124751	KRBA2	KRAB-A domain containing 2	DOWN	-0.41	0.007993576
130535	KCTD18	potassium channel tetramerization domain containing 18	DOWN	-0.477	0.006773075
134957	STXBP5	syntaxin binding protein 5 (tomosyn)	DOWN	-0.43	0.002545894
135112	NCOA7	nuclear receptor coactivator 7	DOWN	-0.515	0.000858682
140772	SMCR6	Smith-Magenis syndrome chromosome region, candidate 6 (non-protein coding)	DOWN	-0.578	0.000607463
140876	FAM65C	family with sequence similarity 65, member C	DOWN	-0.468	0.003031302
144165	PRICKLE1	prickle homolog 1	DOWN	-0.425	0.000553181
145757	#N/A	#N/A	DOWN	-0.414	0.009012792

147650	SPACA6P	sperm acrosome associated 6, pseudogene	DOWN	-0.417	0.002137885
151246	SGOL2	shugoshin-like 2 (S. pombe)	DOWN	-0.51	0.003302612
157869	SBSPON	somatomedin B and thrombospondin, type 1 domain containing	DOWN	-0.434	0.002123089
160418	TMTC3	transmembrane and tetratricopeptide repeat containing 3	DOWN	-0.5	0.00030785
163782	KANK4	KN motif and ankyrin repeat domains 4	DOWN	-0.466	0.001994161
164633	CABP7	calcium binding protein 7	DOWN	-0.575	0.002133314
200576	PIKFYVE	phosphoinositide kinase, FYVE finger containing	DOWN	-0.498	0.00440012
200894	ARL13B	ADP-ribosylation factor-like 13B	DOWN	-0.588	0.000200542
201562	HACD2	3-hydroxyacyl-CoA dehydratase 2	DOWN	-0.486	0.002185737
205428	C3orf58	chromosome 3 open reading frame 58	DOWN	-0.426	0.009664412
205717	USF3	upstream transcription factor family member 3	DOWN	-0.421	0.005522381
219790	RTKN2	rhotekin 2	DOWN	-0.518	0.000589524
221895	JAZF1	JAZF zinc finger 1	DOWN	-0.489	0.002278842
222161	DKFZP586I1420	uncharacterized protein DKFZp586I1420	DOWN	-0.621	0.000181541
253959	RALGAPA1	Ral GTPase activating protein, alpha subunit 1 (catalytic)	DOWN	-0.424	0.001757159
256691	MAMDC2	MAM domain containing 2	DOWN	-0.494	0.007924616
259266	ASPM	abnormal spindle microtubule assembly	DOWN	-0.558	0.006899825
284900	TTC28-AS1	TTC28 antisense RNA 1	DOWN	-0.601	0.000624918
285489	DOK7	docking protein 7	DOWN	-0.555	0.001259427
285671	RNF180	ring finger protein 180	DOWN	-0.416	0.003888308
286205	SCAI	suppressor of cancer cell invasion	DOWN	-0.416	0.001259632
286272	LOC286272	uncharacterized LOC286272	DOWN	-0.512	0.009404411
286676	ILDR1	immunoglobulin-like domain containing receptor 1	DOWN	-0.475	0.000768698
339766	MROH2A	maestro heat-like repeat family member 2A	DOWN	-0.409	0.009308487
340335	LOC340335	uncharacterized LOC340335	DOWN	-0.617	0.006110617
388677	NOTCH2NL	notch 2 N-terminal like	DOWN	-0.464	0.00994507
389792	IER5L	immediate early response 5-like	DOWN	-0.608	0.001346449
399761	BMS1P5	BMS1 ribosome biogenesis factor pseudogene 5	DOWN	-0.451	0.005860164
401261	LOC401261	zinc finger protein ZIC 5	DOWN	-0.408	0.003151064

440570	LOC440570	uncharacterized LOC440570	DOWN	-0.935	0.003725435
441273	SPDYE2	speedy/RINGO cell cycle regulator family member E2	DOWN	-0.459	0.006771927
594839	SNORA33	small nucleolar RNA, H/ACA box 33	DOWN	-0.512	0.002197549
644285	LOC644285	uncharacterized LOC644285	DOWN	-0.449	0.005576684
646603	C4orf51	chromosome 4 open reading frame 51	DOWN	-0.422	0.00651456
647946	MIR924HG	MIR924 host gene	DOWN	-0.535	0.001378907
653082	ZDHHC11B	zinc finger, DHHC-type containing 11B	DOWN	-0.447	0.002654502
728061	LOC728061	hCG2003663	DOWN	-0.422	0.004285259
728190	NUTM2A-AS1	NUTM2A antisense RNA 1	DOWN	-0.528	0.001593378
728215	FAM155A	family with sequence similarity 155, member A	DOWN	-0.516	0.002194204
728577	CNTNAP3B	contactin associated protein-like 3B	DOWN	-0.497	0.000957546
728833	FAM72D	family with sequence similarity 72, member D	DOWN	-0.666	0.002669839
730183	LOC730183	uncharacterized LOC730183	DOWN	-0.598	0.006189425
100113407	TMEM170B	transmembrane protein 170B	DOWN	-0.515	0.004794275
100130428	LOC100130428	IGYY565	DOWN	-0.44	0.002591178
100131541	LOC100131541	uncharacterized LOC100131541	DOWN	-0.409	0.004144216
100216546	LINC01004	long intergenic non-protein coding RNA 1004	DOWN	-0.485	0.002371638
100505385	IQCJ-SCHIP1	IQCJ-SCHIP1 readthrough	DOWN	-0.545	0.00587984
100505494	ANKRD10-IT1	ANKRD10 intronic transcript 1	DOWN	-0.505	0.007066242
100996442	LOC100996442	uncharacterized LOC100996442	DOWN	-0.477	0.000984262
101930159	#N/A	#N/A	DOWN	-0.41	0.0043267
101930489	#N/A	#N/A	DOWN	-0.408	0.006046534
