# Supplementary material for: RUNX3 methylation drives hypoxia-induced cell proliferation and antiapoptosis in early tumorigenesis
Source: Cell Death Differ. 2020 Oct 28;28(4):1251–69. doi: 10.1038/s41418-020-00647-1 (PMC8027031; doi:10.1038/s41418-020-00647-1)
Supplement: Supplementary file 8 — Table S3 [file 41418_2020_647_MOESM8_ESM.pdf]

Table S3. siRNA Sequences

Genes	Sequences
Scrambled siRNA	5′-CAUUCACUCAGGUCAUCAGTT-3′
G9a siRNA #1	5′-AAGCUCUAACUGAACAACUAA-3′
G9a siRNA #2	5′-CACCAUGAACAUCGAUCGCAA-3′.
RUNX3 siRNA	5′-GUGAUGGCAGGCAAUGACGAGAACU-3′
