# Supplementary material for: RUNX3 methylation drives hypoxia-induced cell proliferation and antiapoptosis in early tumorigenesis
Source: Cell Death Differ. 2020 Oct 28;28(4):1251–69. doi: 10.1038/s41418-020-00647-1 (PMC8027031; doi:10.1038/s41418-020-00647-1)
Supplement: Supplementary file 9 — Table S4 [file 41418_2020_647_MOESM9_ESM.pdf]

Table S4. Primer Sets for Quantitative Real-Time PCR

Genes	Sequences	
BCL2L11 (ChIP)	Forward	5′-TGGGTAAGAGGCAGTTGACG-3′
	Reverse	5′-TTGACACATCCTCCATTCCCA-3′
BCL2L11	Forward	5′-GCCAGGCCTTCAACCCATAT-3′
	Reverse	5′-TCCAATACGCCGCAACTCTT-3′
MX1 (ChIP)	Forward	5′-GCTCTCCAAAGCTCACCAGT-3′
	Reverse	5′-TTGGTCCTCAAGGTCAAGGC-3′
MX1	Forward	5′-GATGGCATTCTGGGCTTTAT-3′
	Reverse	5′-AGTGGAGAGGCAAGGTCAGT-3′
TRIM22 (ChIP)	Forward	5′-ACAGAGAAAACACAGAGCTCCA-3′
	Reverse	5′-ACCAGAAAACGTGGGCCAAT-3′
TRIM22	Forward	5′-AGGAAAAGCTGCAGGTAGCC-3′
	Reverse	5′-TGGATATAATTCTTCCAGGCGG-3'
NLRP3 (ChIP)	Forward	5′-AAGCTATCCTCCCGCCTCTT-3′
	Reverse	5′-TGGGTGACAAGAGCAAGACT-3′
NLRP3	Forward	5′-GGCAACACTCTCGGAGACAA-3′
	Reverse	5′-GGAAAGATCCCAGCAGCAGT-3′
IL1β (ChIP)	Forward	5′-AACCGAGACACCAGCAAAGT-3′
	Reverse	5′-GAGGCAAAGGAGGGTGTTCC-3′
IL1β	Forward	5′-TGGCTTATTACAGTGGCAATGA-3′
	Reverse	5'-GGTCGGAGATTCGTAGCTGG-3'
