# Supplementary material for: RUNX3 methylation drives hypoxia-induced cell proliferation and antiapoptosis in early tumorigenesis
Source: Cell Death Differ. 2020 Oct 28;28(4):1251–69. doi: 10.1038/s41418-020-00647-1 (PMC8027031; doi:10.1038/s41418-020-00647-1)
Supplement: Supplementary file 10 — RUNX3-meth-Supplementary-CDD-R [file 41418_2020_647_MOESM10_ESM.docx]

**Supplementary Information**

**RUNX3 methylation drives hypoxia-induced cell proliferation and antiapoptosis in early tumorigenesis**

Sun Hee Lee, Do Young Hyeon, Soo-Hyun Yoon, Ji-Hak Jeong, Saeng-Myung Han, Ju-Won Jang, Minh Phuong Nguyen, Xin-Zi Chi, Sojin An, Kyung-gi Hyun, Hee-Jung Jung, Ji-Joon Song, Suk-Chul Bae, Woo-Ho Kim, Daehee Hwang, and You Mie Lee

**Supplemental Figure Legends**

**Figure S1, Related Figure 1. Negative correlation between expression of G9a and expression of RUNX3 protein.**

(**A**) Semi-quantitative RT-PCR and (**B**) densitometric analysis were used to examine expression RUNX3 mRNA in gastric cancer cells exposed to hypoxia for the indicated times. (**C**) Co-immunoprecipitation (IP) assay of RUNX3 and G9a in SNU484 cells. Whole cell lysates (400 µg protein) obtained under normoxic (N) or hypoxic conditions and immunoprecipitates with indicated antibodies were analyzed by immunoblotting with anti-G9a or anti-RUNX3 antibodies. (**D**) Nuclear (Nucl) or cytoplasmic (Cyto) extracts (400 µg protein) under normoxic (N) and hypoxic (H) conditions for 8 h were analyzed by IP/IB analysis. Lamin A/C and α-tubulin antibodies were used for a nucleus and a cytosolic marker, respectively. Immunoprecipitates with anti-G9a antibody were analyzed by immunoblotting with anti-RUNX3 or anti-G9a antibodies. (**E**) Proportion on the binding from Figure R1B was estimated by ImageJ software. (**F**) Immunofluorescence (IF) analysis of RUNX3 and α-Tubulin in SNU484 cells under normoxia or hypoxia for 8 h. Nuclei were counterstained with DAPI. Scale bar = 10 μm. (**G**) Immunofluorescence analysis of RUNX3 and G9a expression in human cancer tissue section. Nuclei were counterstained with Harris modified hematoxylin.

**Figure S2, Related Figure 5. Peak intensity changes in the regulatory regions.**

(**A-C**) Distributions (top) and cumulative density functions (bottom) of log_2_-fold-changes of consensus peak intensities in up- and down-regulated genes and non-DEGs were obtained in downstream regulatory region (**A**), 1^st^ intron (**B**), and other introns (**C**).

**Figure S3, Related Figure 6. Increased promoter binding of RUNX3.**

(**A**) Relative colony counts of SNU484 cells expressing WT and mutant RUNX3 proteins under normoxia (N) or hypoxia (H). Data are expressed as the mean ± S.D. (n = 3). *, p < 0.05 (normoxia) and ^#^, p < 0.05 (hypoxia) by one-way ANOVA with Tukey’s posthoc corrections. (**B**) Read distributions in the promoters of the three representative RUNX3 target genes. Chromosome number, and the coordinates, and gene structures are shown. TSS, transcription start site.

**Figure S4, Related Figure 6. RUNX3 overexpression inhibits tumor growth.**

Tumor sizes measured by *in vivo* xenograft assays using MKN1 cells overexpressing the indicated constructs. Tumor size was measured at the indicated days. Data are shown as the mean ± S.D. n = 8 mice/group. *, p < 0.05 by Student’s t-test.

**Figure S5, Related Figure 7. Clinical relevance of RUNX3 target genes in human gastric cancers.**

Comparison of survival for two groups of patients in TCGA and ACRG gastric cancer cohorts with higher and lower expression levels of RUNX3 (top and bottom 25% of patients with the highest and lowest mRNA expression levels, respectively, in each cohort).
